# Genome-wide CRISPR-cas9 knockout screening identifies GRB7 as a driver for MEK inhibitor resistance in KRAS mutant colon cancer

**DOI:** 10.1038/s41388-021-02077-w

**Published:** 2021-10-30

**Authors:** Chune Yu, Dan Luo, Jing Yu, Min Zhang, Xiaobo Zheng, Guangchao Xu, Jiaxin Wang, Huiling Wang, Yufei Xu, Ke Jiang, Jie Xu, Xuelei Ma, Jing Jing, Hubing Shi

**Affiliations:** 1 Laboratory of Integrative Medicine, Clinical Research Center for Breast, State Key Laboratory of Biotherapy, West China Hospital, Sichuan University and Collaborative Innovation Center, Chengdu, Sichuan 610041 China; 2 Department of Immunology, School of Basic Medical Sciences, Chengdu Medical College, Chengdu, Sichuan 610500 China; 3 Department of Plastic Surgery, Affiliated Hospital of Zunyi Medical University, Zunyi, Guizhou 563000 China; 4 Department of Head and Neck, Sun Yat-sen University Cancer Center, State Key Laboratory of Oncology in South China, Collaborative Innovation Center for Cancer Medicine, Guangzhou, 510000 China; 5 Division of Gastroenterology and Hepatology, Renji Hospital, School of Medicine, Shanghai Jiao Tong University, 145 Middle Shandong Road, 200001 Shanghai, China; 6 Department of Biotherapy, State Key Laboratory of Biotherapy, Cancer Center, West China Hospital, Sichuan University, Chengdu, Sichuan 610041 China

**Keywords:** Cancer therapeutic resistance, Targeted therapies, Colorectal cancer

## Abstract

Targeting the KRAS pathway is a promising but challenging approach for colorectal cancer therapy. Despite showing potent efficacy in BRAF-mutated melanoma, MEK inhibitors appeared to be tolerated by colorectal cancer cells due to their intrinsic compensatory signaling. Here, we performed genome-wide CRISPR/Cas9 screening in the presence of MEK inhibitor to identify genes that are synthetically lethal with MEK inhibition in CRC models harboring KRAS mutations. Several genes were identified as potential functional drivers, which were significantly enriched in the GRB7-mediated RTK pathway. Loss-of-function and gain-of-function assays validated that GRB7 potently rendered CRC cells primary resistance to MEK inhibitors through the RTK pathway. Mass spectrum analysis of GRB7 immunoprecipitates revealed that PLK1 was the predominant interacting kinase of GRB7. Inhibition of PLK1 suppressed downstream signaling of RTK, including FAK, STAT3, AKT, and 4EBP1. The combination of PLK1 and MEK inhibitors synergistically inhibited CRC cell proliferation and induced apoptosis in vitro and in vivo. In conclusion, we identified GRB7-PLK1 as a pivotal axis mediating RTKs, resulting in MEK inhibitor tolerance. PLK1 is therefore a promising target for synergizing MEK inhibitors in the clinical treatment of CRC patients harboring KRAS mutations.

## Introduction

Colorectal cancer (CRC) is one of the most common malignancies, with the third highest incidence and death rate in both women and men in the United States [1]. Approximately 40% of CRCs harbor oncogenic KRAS mutations, mainly G12D, G12V, and G13D, which can sustainedly activate the MAPK signaling pathway [2, 3]. Given the fact that KRAS mutations are often associated with poor prognosis in CRC patients [4], therapeutic regimens targeting KRAS or its signaling are regarded as one of the most desired strategies in clinics [5]. Although two small molecules, AMG510 and MRTX849, which target KRAS G12C have entered clinical trials, these inhibitors produced less profound activity in CRC than non-small cell lung cancer patients [6–10]. In addition, the KRAS G12C mutation accounts for only one to three percent of all CRC patients, which largely limits the potential populations who could benefit [2, 11]. Thus, a strategy that directly targets a broad spectrum of KRAS mutations remains challenging.

Since RAS can activate the MAPK pathway, which consists of a linear kinase cascade, blockade of downstream kinases, vertical repression of MEK, has previously been considered a promising strategy [12]. However, the results of clinical trials with MEK inhibitor (MEKi) were disappointing in CRC patients [13, 14]. The negative clinical outcome was attributed to the fact that blockading MEK released the negative feedback regulation of RTKs [15–17]. Upon activation, RTKs might reactivate the MAPK pathway or activate redundant pathways to confer resistance to MAPK inhibitors. Notably, multiple RTKs are attributed to this process, implying that attenuation of this adaptive resistance by targeting a single RTK may not be an effective strategy [8]. Therefore, identifying pivotal mediators in the negative feedback loop of the RTK pathway and providing combination therapy to overcome MEKi resistance are urgently needed.

Multiple high-throughput genome-wide screening approaches have been applied to unbiasedly identify critical genes that contribute to drug resistance in human cancers. Previously, RNA interference (RNAi) screening with shRNA library to knockdown targeted genes has been widely used [18, 19]. However, inefficient gene knockdown and off-target effects limited their applications [20]. Recently, the CRISPR-Cas9 library system with innovations in genome editing technology has provided an alternative way to overcome these limitations. It has been used to identify genes that are essential for cancer cell survival, proliferation, and targeted drug resistance in vitro and in vivo [21–24].

Therefore, we performed genome-wide CRISPR/Cas9 knockout screening to identify the pivotal oncogenes contributing to overcoming MEKi resistance in CRC cells. Further, we performed co-targeting of MEK and PLK1 kinases to investigate its effect on CRC both in vitro and in vivo. Our findings will not only help clarify the mechanism of primary resistance to MEK-targeted therapy in CRC, but also provide a combinatorial regimen for clinical treatment.

## Results

### RTK pathway mediates MEKi resistance in KRAS mutant colon cancer

**Fig. 1:**
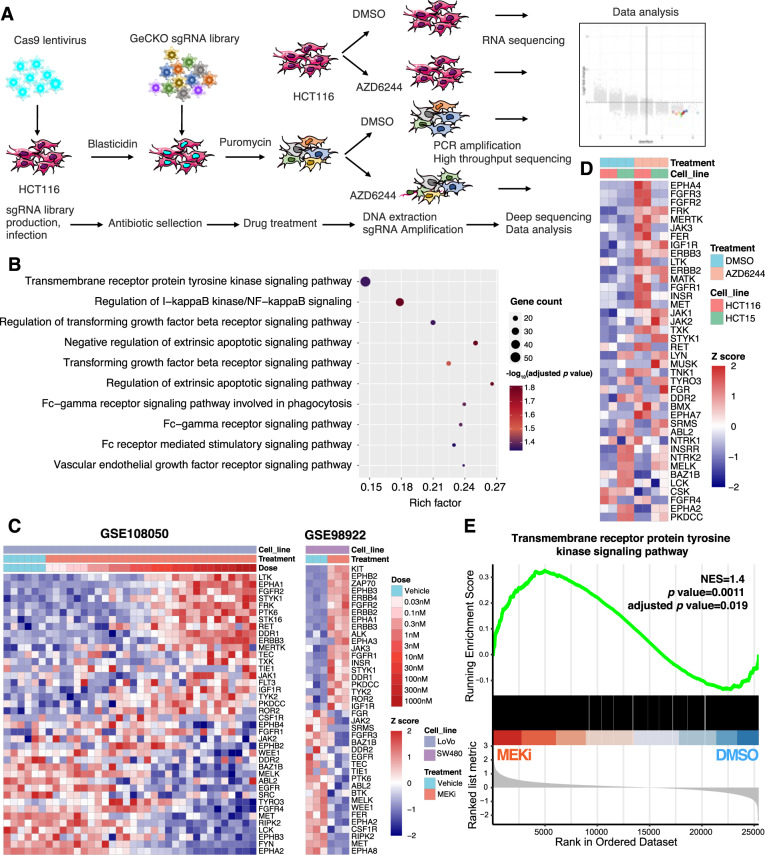
CRISPR library screening identifying the involvement of the RTKs pathway in MEKi resistance in KRAS mutation colon cancer cells. **A** Outline of genome-wide CRISPR/Cas9 knockout library screening in CRC cells. **B** Gene Ontology (GO) enrichment analysis of significantly downregulated genes in HCT116 cells treated with AZD6244. The Benjamini & Hochberg (BH) method was used for multiple comparison. **C** Heatmap of RTK genes in CRC cells treated with MEKi compared with DMSO control in GEO datasets. **D** Heatmap of RTKs genes in CRC cells treated with AZD6244 for 7 days. **E** GSEA analysis of the transmembrane receptor protein tyrosine kinase signaling pathway gene set in CRC cells treated with or without MEKi; datasets were from (**C**) and (**D**). *p* value and adjusted *p* value were determined by permutation test and Benjamini & Hochberg (BH) method, respectively. NES normalized enrichment score.

To identify the critical driver genes involved in MEKi resistance, we designed a combination strategy with genome-wide CRISPR/Cas9 knockout library screening and transcriptomic analysis of KRAS-mutated cell lines (Fig. 1A). The model cell line HCT116 was selected, which exhibited a relatively medium drug tolerance capability for MEKi in a short-term cell proliferation assay. The IC50 was around 3.6 µM (Supplementary Fig. S1A). To maintain an approximate 500× sgRNA coverage, 200 million cells stably expressing Cas9 were infected with the human GeCKO library two-vector lentivirus system. The single gene knockout in each cell was strictly controlled by adjusting the infective MOI to close to 0.3. After puromycin selection, cells were treated with DMSO or AZD6244 for 7 days. Subsequently, genomic DNA encompassing sgRNA was extracted and assessed by PCR amplification and high-throughput sequencing (Supplementary Table S1). The quality and selective enrichment number of sgRNA reads showed comparable patterns between the DMSO-treated group and the AZD6244-treated group, indicating that no obvious bias selection was observed in the screening process (Supplementary Fig. S1B, C). Theoretically, genes whose deletion causes MEKi lethality should preferably be observed in DMSO-treated cells as opposed to AZD6244-treated cells, thus providing a novel combination treatment. Considering the off-target effects of sgRNA, we selected genes with at least three downregulated sgRNAs in AZD6244-treated cells, as preliminary candidates. In parallel, we profiled the transcriptome of HCT116 cells in the presence or absence of AZD6244 for 7 days. We assumed that the genes driving resistance were transcripted effectively in AZD6244-treated cells. Thus, the preliminary identified genes with a TPM value of no less than 10 (moderate expression level) were identified as confident candidate genes [25]. Based on this strategy, we identified a series of sgRNAs targeting 1846 genes, which were significantly depleted in AZD6244-treated cells compared to DMSO-treated cells (Supplementary Fig. S1D). The results of Gene Ontology (GO) enrichment analysis showed that these genes were predominantly distributed in some pathways, such as the transmembrane receptor protein tyrosine kinase signaling (RTK pathway), the NF-κB pathway, TGF-β pathway, and the regulation of extrinsic apoptotic pathway (Fig. 1B). Transcriptomic analysis of CRC cells revealed that the expression patterns of RTK genes were significantly disrupted upon MEKi treatment (Fig. 1C, D). In line with this cellular sequencing data, similar phenomena were observed in another independent experiment of CRC organoids treated with MEKi (Supplementary Fig. S1E). Furthermore, gene set enrichment analysis (GSEA) also demonstrated that expression of RTK pathway genes was significantly upregulated in CRC cells treated with MEKi (Fig. 1E). To further confirm this result, we performed the western blot analysis of proteins in the RTK pathway upon MEKi treatment (Supplementary Fig. S1F). Phosphorylation levels of EGFR, MET, FAK, STAT3, and AKT were significantly upregulated by AZD6244 treatment in HCT116 and SW480 cells. These data indicate that the RTK pathway plays a pivotal role in MEKi resistance. We then focused on investigating the genes that were enriched in the RTK pathway.

### GRB7 is the pivotal member of the RTK pathway-mediated MEKi resistance

**Fig. 2:**
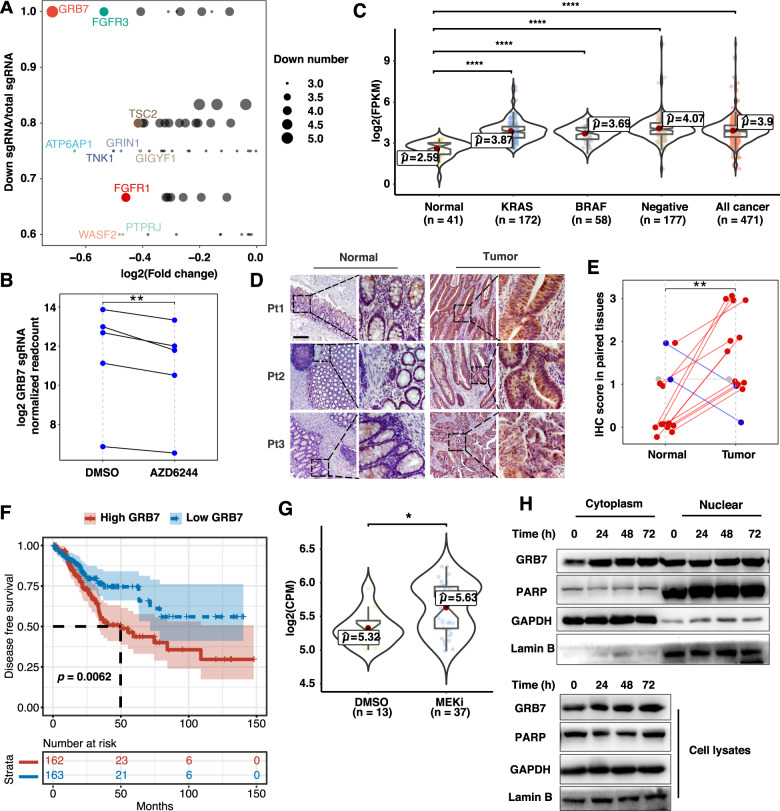
GRB7 was the determent in the RTK pathway for MEKi resistance. **A** Scatter plot showing the genes that were enriched in the RTK pathway. The top ten downregulated genes based on log2(fold change) are represented. The size of the point represents the number of identified downregulated sgRNA sequences for each gene. **B** The sgRNAs targeting GRB7 were consistently depleted in AZD6244-treated cells. The Student’s *t* test was used to determine the *p* value. **C** Boxplot of GRB7 expression in normal and tumor tissues of colon cancer patient samples with or without the BRAF mutation and KRAS mutations from TCGA-COAD. The Wilcoxon test was used to determine the *p* value. **D**, **E** Representative immunohistochemical images of GRB7 staining in normal and tumor tissues from colon cancer patients. Scale bar, 200 μM. **E** Quantification of (**D**). Data represented as mean ± SD (*n* = 14). The Wilcoxon test was used to determine the *p* value. **F** Kaplan-Meier survival analysis of the association of GRB7 levels with disease-free survival (DFS) of colon cancer patients. Patients were divided into low or high groups according to the expression of GRB7. The log-rank test was used to determine the *p* value. **G** GRB7 mRNA expression was increased in AZD6244-treated cells. Datasets were from Fig. 1C, D. The Wilcoxon test was used to determine the *p* value. Batch effects between different datasets were removed by ComBat. **H** Cell cytoplasm/nucleus fractionation and western blot analysis to show GRB7 translocation with AZD6244 treatment. HCT116 cells treated with AZD6244 were fractionated into nuclear and cytoplasmic fractions at the indicated time points. *p < 0.05, **p < 0.01, ***p < 0.001, or ****p < 0.0001; TCGA-COAD The Cancer Genome Atlas Colon Adenocarcinoma.

Among the genes involved in the RTK pathway, growth factor receptor-bound protein 7 (GRB7) was identified as the most negatively selected gene after AZD6244 treatment (Fig. 2A). Five out of six sgRNAs targeting GRB7 were consistently decreased in AZD6244-treated cells, while the remaining sgRNA was not detectable (Fig. 2B). The results indicated that GRB7 knockout caused a synthetic lethality effect along with MEKi, implying that GRB7 might confer resistance to MAPK inhibition. Importantly, upregulation of GRB7 was detected in CRC tissues (from TCGA data) with KRAS mutations, BRAF mutation, and double wild-type genotypes compared to that in normal colon tissues, suggesting an intrinsic function of GRB7 in tumor tissue (Fig. 2C). To validate these results, we analyzed GRB7 levels in our collected biopsies by IHC (Fig. 2D). Consistent with the mRNA expression results, upregulation of GRB7 at the protein level was observed in 11 out of 14 patient-matched CRC biopsies (Fig. 2D, E). By analyzing the TCGA database, we found that high levels of GRB7 were significantly associated with poor outcomes in CRC patients (Fig. 2F). Interestingly, analysis of a series of cancer types that harbored aberrant RAS and/or RAF mutations as oncogenic drivers indicated that the expression of GRB7 in lung carcinoma and pancreatic cancer was much higher than that in melanoma (Supplementary Fig. S2). This was consistent with the fact that melanoma with BRAF or NRAS mutations were highly dependent on the MAPK pathway and showed a favorable response to MAPK inhibitors [26, 27]. It also implied that GRB7 might potentially mediate drug tolerance in these cancer types. To further investigate the role of GRB7 in MEKi resistance, we combined the datasets of GSE108050, GSE98922, and ours, then dissected the transcriptome profiling data of CRC cells in the presence or absence of MEKi (Fig. 2G). Significant upregulation of GRB7 was observed in multiple CRC cell lines with MEK inhibition, implying that the GRB7 signaling axis might be activated by MEKi treatment. To test this hypothesis, we investigated the subcellular localization of GRB7 following MEKi treatment. GRB7 was significantly upregulated in cytosolic fractions upon MEKi treatment from 24 to 72 h (Fig. 2H). Collectively, these results demonstrate that GRB7 may play a pivotal role in MEKi resistance.

### GRB7 mediates MEKi resistance in KRAS-mutated CRC cells

**Fig. 3:**
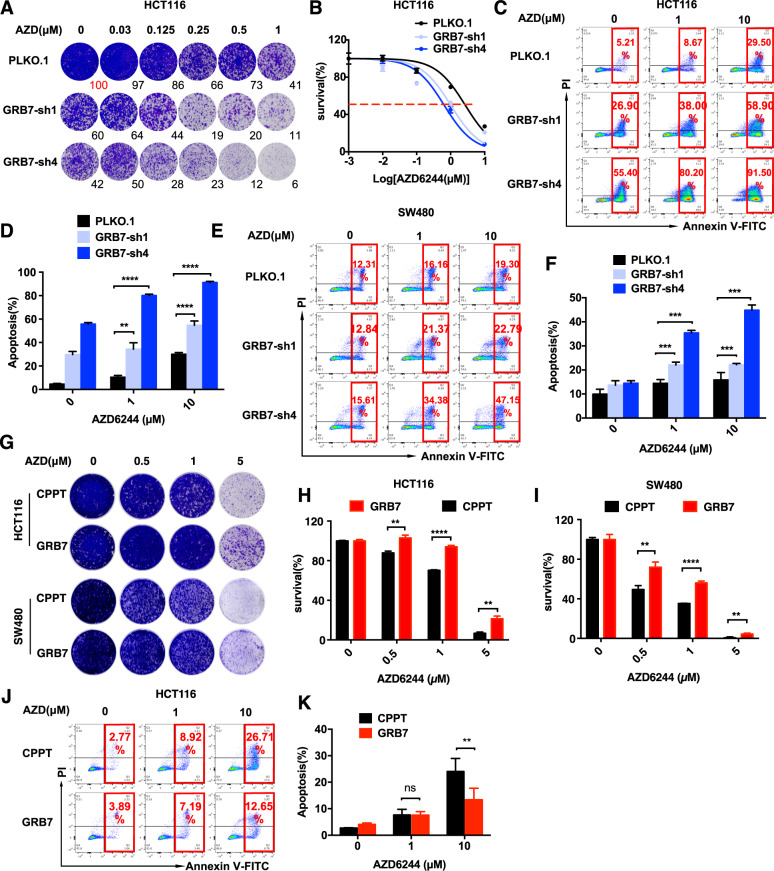
GRB7 is sufficient for MEKi resistance in KRAS mutation colon cancer cells. **A** Clonogenic assay showing that knockdown of GRB7 with shRNAs enhanced response to MEKi. **B** Short-term survival curves identifying that knockdown of GRB7 increased the sensitivity of MEKi. Results are shown relative to DMSO-treated controls (mean ± SD, *n* = 5; dashed line, 50% inhibition). **C**–**F** Knockdown of GRB7 significantly induced apoptosis upon AZD6244 treatment in HCTT16 (**C**, **D**) and SW480 (**E**, **F**). Cells were treated with AZD6244 for 48 h (HCT116) or 24 h (SW480). Data represented as mean ± SD (*n* = 3). **G**–**I** Clonogenic assay showing that GRB7 overexpression conferred resistance to AZD6244. (**H**) Quantification of HCT116, (**I**) Quantification of SW480. Data represented as mean ± SD (*n* = 3). **J**, **K** Induction of anti-apoptosis upon overexpression of GRB7 in HCT116. **K** Quantification of (**J**). Data represented as mean ± SD (*n* = 3). *p < 0.05, **p < 0.01, ***p < 0.001, or ****p < 0.0001.

To validate the function of GRB7 in terms of driving primary resistance to MAPKi using an orthogonal approach, we evaluated CRC cell proliferation in the presence of MEKi with shRNA-mediated perturbation of GRB7. The efficiencies of shRNAs were determined by qPCR, and optimized sequences were used in the functional experiments (Supplementary Fig. S3A). Both the long-term clonogenic assay and short-term MTT assay indicated that cell proliferation was significantly inhibited in HCT116, SW480, and LS174T cell lines with GRB7 knockdown in the presence of AZD6244 (Fig. 3A, B, and Supplementary Fig. S3B, C). Moreover, GRB7 knockdown synergistically induced cell apoptosis with AZD6244 treatment for 24 and 48 h in CRC cell lines (Fig. 3C–F, Supplementary Fig. S3D, E, and Table S2). Similar to AZD6244, GSK1120212, another MEKi approved in the clinic, combined with GRB7 knockdown, induced supra-additive apoptosis in CRC cells (Supplementary Fig. S3F). Apoptosis analysis in A549 (lung cancer) and CFPAC-1 (pancreatic cancer) cells was performed to determine whether GRB7 mediates MEKi resistance in other RAS-driven cancer types. The mechanism of primary resistance in these cells was reported to be activation of the RTK-mTOR signaling pathway [28]. The results showed that GRB7 knockdown further promoted apoptosis when combined with AZD6244 in A549 and CFPAC-1 cells (Supplementary Fig. S3G).

In addition to the loss-of-function validation, we also tested the gain of function by overexpressing GRB7 in CRC model cell lines. The proliferation of HCT116 and SW480 cells, which were infected with lentivirus encoding FLAG-tagged GRB7, was evaluated in the presence of the MEKi (Supplementary Fig. S4A). The results of the clonogenic assay demonstrated that overexpression of GRB7 significantly promoted resistance to AZD6244 (Fig. 3G–I). In addition, overexpression of GRB7 also relieved cell apoptosis induced by high doses of AZD6244 in HCT116 cells (Fig. 3J, K). Inspired by these results, we sought to investigate whether GRB7 overexpression could also confer MEKi resistance in CRC cells with the BRAF mutation, another genetic lesion leading to sustained activation of the MAPK pathway [29]. Similarly, ectopic expression of GRB7 in HT29 and WIDR cells, which harbor the ^V600E^BRAF mutation, also significantly promoted resistance to AZD6244 in the long-term clonogenic assay (Supplementary Fig. S4B–E). Taken together, these results demonstrate that GRB7 mediates MEKi resistance in CRC cells with KRAS or BRAF mutations.

### GRB7 mediates the activation of the RTK downstream pathway to render MEKi resistance in CRC cells

**Fig. 4:**
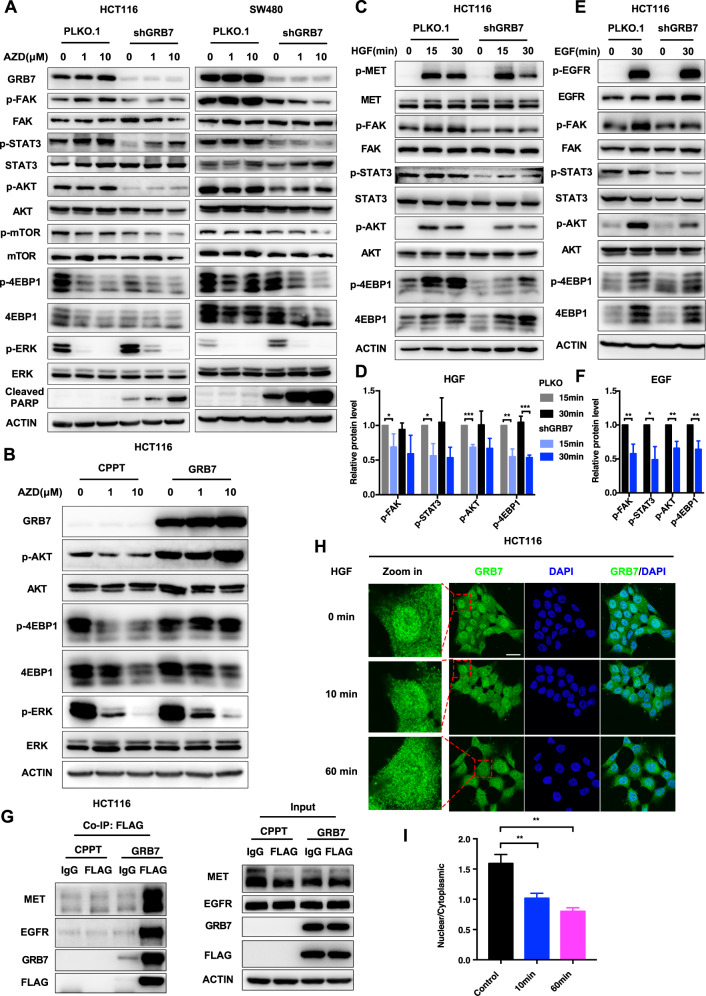
GRB7 conferred resistance to MEKi through regulation of downstream RTK pathway. **A** Western blot analysis showed that combinational inhibition of GRB7 and MEK suppressed the downstream effectors in RTK pathway. HCT116 and SW480 were infected with shRNA4 targeting GRB7 or vector control. After infection, cells were treated with AZD6244 for 24 h. Cell lysates were made for immunoblot analysis with antibodies indicated. ACTIN is as loading control. **B** Overexpression of GRB7 activated RTK pathway. HCT116 were ectopically expressed GRB7 or vector control. After infection, cells were treated with AZD6244 for 24 h. Cell lysates were made for immunoblot analysis with antibodies indicated. ACTIN is as loading control. **C**–**F** Knockdown of GRB7 impaired HGF/EGF-dependent pathway. HCT116 were infected with shRNA4 targeting GRB7 or vector control. After infection, cells were treated with 20 ng/mL HGF(**C**) or EGF (**E**) for the indicated time. Cell lysates were made for immunoblot with antibodies indicated. ACTIN is as loading control. **D** Quantification of (**C**). **F** Quantification of (**E**). **G** GRB7 interacted with RTKs directly. Cell lysates from GRB7 expressing or vector control cells were subjected to immunoprecipitation with Flag antibody or immunoglobulin G control. The immunoprecipitants (left) or cell lysates (right) were then blotted with the indicated antibodies. **H** Representative immunofluorescence images of GRB7 in HCT116 treated with 20 ng/mL HGF for indicated time. Scale bar, 20 μM. **I** Quantification of (**H**). *p < 0.05, **p < 0.01, ***p < 0.001, or ****p < 0.0001.

To investigate whether GRB7 confers resistance to MEKi by mediating the RTK pathway, we characterized the activation status of effectors within this pathway, including FAK, STAT3, AKT, mTOR, 4EBP1, and ERK in CRC cell lines with or without GRB7 knockdown in the presence of MEK inhibitors (Fig. 4A and Supplementary Fig. S4F). The western blot results showed that GRB7 knockdown significantly attenuated the phosphorylation levels of FAK, STAT3, AKT, mTOR, and 4EBP1, although some of them, such as FAK and STAT3, were compensatively evoked by MEK inhibition. Interestingly, GRB7 knockdown slightly increased the level of p-ERK, indicating compensatory feedback signaling. In line with previous apoptosis assays, the level of cleaved PARP induced by GRB7 knockdown was further promoted by MEKi in HCT116 and SW480 cells (Fig. 4A and Supplementary Fig. S4F). In parallel, we ectopically overexpressed GRB7 and detected the downstream activation of RTK. The western blot results showed that the phosphorylations of AKT and 4EBP1 increased upon GRB7 overexpression in CRC cells (Fig. 4B and Supplementary Fig. S4G). These results suggest that GRB7 potently mediates the activation of the RTK downstream pathway. To further investigate whether GRB7 is essential for the activation of RTK pathways, we challenged RTKs with their ligands and probed the activation of downstream effectors upon perturbation of GRB7. It has previously been reported that c-MET and EGFR were the predominant activated RTKs upon AZD6244 treatment in HCT116 cells [17], therefore we chose their ligands HGF and EGF to stimulate c-MET and EGFR, respectively (Fig. 4C–F). The phosphorylation levels of FAK, AKT, STAT3, and 4EBP1 were reversely attenuated by GRB7 knockdown which was upregulated by stimulation with HGF or EGF. These results further strengthen the conclusion that GRB7 mediates activation of the RTK pathway.

Since GRB7 contains an SH2 domain, we speculated that GRB7 mediates the RTK pathway by transducing signaling through direct interaction. Given the heterogeneous and dynamic expression pattern of RTK, we chose to probe the interaction between GRB7 and two representative receptors, EGFR and MET. The results of the co-IP experiment indicated that GRB7 formed a complex with EGFR and MET in HCT116 cells (Fig. 4G). To further confirm this direct interaction between RTKs and GRB7, the subcellular distribution of GRB7, upon stimulation with HGF, a MET ligand, was investigated. Similar to EGF [30], HGF stimulation facilitated the export of GRB7 from the nucleus to the cytoplasm (Fig. 4H, I), which might be a common approach to the regulation of GRB7 signaling by growth factors. Collectively, these data strengthen the conclusion that GRB7 mediates the activation of the RTK signaling pathway by direct interaction, which sufficiently confers MEKi resistance in CRC cells.

### GRB7 mediates the RTK pathway through PLK1 recruitment

**Fig. 5:**
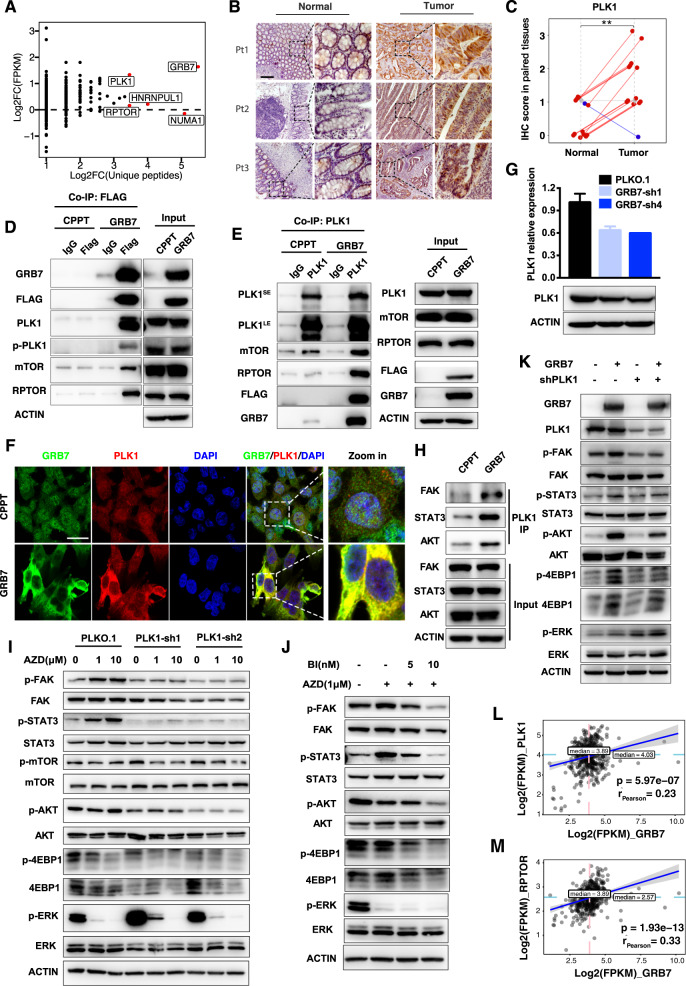
RTK pathway activated by GRB7 through PLK1. **A** Dot plot revealing that PLK1 was the most upregulated protein in colon cancer tissues from TCGA data of the top five GRB7-interacting proteins. The *y*-axis represents the log2 fold change of FPKM between tumor and normal tissues in the TCGA-COAD. The *x*-axis represents the log2 fold change of unique peptides between HCT116 expressing FLAG-GRB7 and control cells by MS. The top five genes are displayed in red according to the unique peptides. **B**, **C** Immunohistochemical images of PLK1 staining in normal and tumor tissues from colon cancer patients. Scale bar, 200 μM. **C** Quantification of (**B**). Data represented as mean ± SD (*n* = 14). The Wilcoxon test was used to determine the *p* value. **D**, **E** GRB7 interacted with PLK1 and RPTOR directly. Cell lysates from HCT116 expressing GRB7 or vector control were subjected to immunoprecipitation with Flag antibody (**D**) or PLK1 antibody (**E**) or immunoglobulin G control. The immunoprecipitants or cell lysates were then blotted with the indicated antibodies. **F** Representative immunofluorescence images for GRB7 and PLK1 in HCT116 expressing GRB7 or vector control. Scale bar, 20 μM. **G** HCT116 cells were infected with shRNAs targeting GRB7 or vector control. Cells were collected for qPCR analysis (top) or cell lysates were made for immunoblot against indicated antibodies (bottom). Data represented as mean ± SD (*n* = 3). **H** GRB7 promoted the interactions between PLK1 and RTK effectors. HCT116 were infected with vector or GRB7 overexpressing plasmid. The cell lysates were immunoprecipitated with an anti-PLK1 antibody, and the precipitates were analyzed by western blot analysis with the indicated antibodies. **I** Western blot analysis showing that combinational inhibition of PLK1 and MEK suppresses the downstream effectors in the RTKs pathway. HCT116 were infected with shRNAs targeting PLK1 or vector control. Actin was used as the control. **J** Western blot analysis of downstream RTK signaling inhibition after AZD6244 with BI-2536 treatment for 24 h in HCT116. **K** Immunoblot analysis of cell lysates, using the indicated antibodies, of HCT116 overexpressing vector alone, FLAG-GRB7, shPLK1, or FLAG-GRB7 plus shPLK1. **L**, **M** Expression correlation analysis of mRNA of GRB7 with PLK1 (**L**), RPTOR (**M**) using RNAseq data of TCGA-COAD samples. The scatter plots show the Pearson correlation of GRB7 with PLK1, RPTOR, and the significance of their correlation is tested by Student’s *t* test. *p < 0.05, **p < 0.01, ***p < 0.001, or ****p < 0.0001.

To further elucidate the molecular mechanism of the GRB7-mediated RTK pathway, we performed immunoprecipitation coupled with mass spectrometry (IP-MS). Potential GRB7-interacting proteins were co-precipitated with anti-FLAG antibody-conjugated beads and subsequently subjected to MS analysis. A total of 57 GRB7-interacting proteins were enriched at least threefold in HCT116 cells expressing FLAG-GRB7 compared to that in control cells (Supplementary Table S3). Since GRB7 mediates intrinsic function in CRC tumor tissues, the proteins mediating GRB7 signaling should be enriched in tumor tissue as opposed to normal tissue. Following this assumption, we found that polo-like kinase-1 (PLK1) was the top-ranked protein, which showed both significant upregulation in CRC tissues compared to normal tissues, and abundant MS identity peptides (Fig. 5A and Supplementary Fig. S5A–D). In line with this assumption, IHC analysis of patient samples showed that PLK1 expression in CRC tumor biopsies was much higher than that in normal tissues (Fig. 5B, C). In addition, regulatory associated protein of mTOR complex 1 (RPTOR), which is a downstream substrate of PLK1 in the GRB7-binding complex was also found. The interactions between PLK1 or RPTOP and GRB7 were validated by co-IP assay. As expected, the results of the co-IP assay with the anti-FLAG antibody showed that GRB7 formed a complex with PLK1 and RPTOR in HCT116 and SW480 cells expressing FLAG-GRB7 (Fig. 5D, Supplementary Fig. S5E). In addition, GRB7 also interacted with phosphorylated PLK1, implying that GRB7 may promote the function of PLK1. A reverse immunoprecipitation assay with PLK1 antibody further confirmed the existence of a complex consisting of GRB7, PLK1, RPTOR, and mTOR (Fig. 5E). Inspired by the interaction between GRB7 and PLK1, we speculated that PLK1 may potentially mediate the RTK-GRB7 signaling pathway. GRB7 was overexpressed in HCT116 cells in order to test this hypothesis. The overexpressed GRB7 was mobilized to the membrane, and a clear co-localization between GRB7 and PLK1 was observed throughout the process (Fig. 5F). Moreover, knockdown of GRB7 with shRNAs downregulated PLK1 by ~40% at both protein and mRNA levels in HCT116 and SW480 cells, suggesting that the regulation was not limited to subcellular localization (Fig. 5G and Supplementary Fig. S5F).

To investigate whether GRB7 regulates the RTK pathway through the recruitment of PLK1, we performed an immunoprecipitation assay with PLK1 antibody in GRB7 overexpressed HCT116 cells and probed the downstream kinases in the RTK pathway (Fig. 5H). The interactions of PLK1 and its downstream kinases (FAK, STAT3, and AKT) were enhanced when GRB7 was overexpressed. Then, PLK1 was knocked down with shRNAs and the downstream effectors in the RTK pathway were evaluated in the presence of AZD6244 (Supplementary Fig. S5G, H). Similar to the results obtained with the GRB7 knockdown, silencing of PLK1 by shRNAs attenuated the phosphorylation levels of FAK, STAT3, AKT, mTOR, and 4EBP1, but slightly increased the phosphorylation of ERK when combined with MEKis (Fig. 5I and Supplementary Fig. S6A, B), suggesting that PLK1 mediated MEKi-induced RTK/GRB7 pathway activation. Consistent with the results of the shRNA assay, inhibition of PLK1 with the specific small molecular inhibitor, BI-2536, impaired the phosphorylation levels of FAK, STAT3, AKT, and 4EBP1 in HCT116 and SW480 cells when combined with AZD6244 (Fig. 5J and Supplementary Fig. S6C). Of note, the upregulations of phosphorylations of FAK, STAT3, AKT, and 4EBP1 induced by GRB7 ectopic expression were reversed by PLK1 knockdown in HCT116 and SW480 cells (Fig. 5K and Supplementary Fig. S6D). These results support the fact that PLK1 is required for the GRB7-mediated RTK pathway. To further prove the relationship between GRB7 and the downstream effectors of the RTK pathway in the clinic, we performed a correlation analysis from TCGA datasets. Significant positive correlations at the mRNA expression level were observed between GRB7 and its downstream components, namely AKT1, AKT2, EIF4EBP1, RPTOR, and PLK1 (Fig. 5L, M and Supplementary Fig. S6E–H).

In summary, our data strongly demonstrated that upregulation of GRB7 conferred MEKi resistance in CRC cells with KRAS mutations by mediating RTK signaling through the recruitment of PLK1.

### Combination inhibition of PLK1 and MEK promotes cell apoptosis in KRAS mutant colon cancer cells

**Fig. 6:**
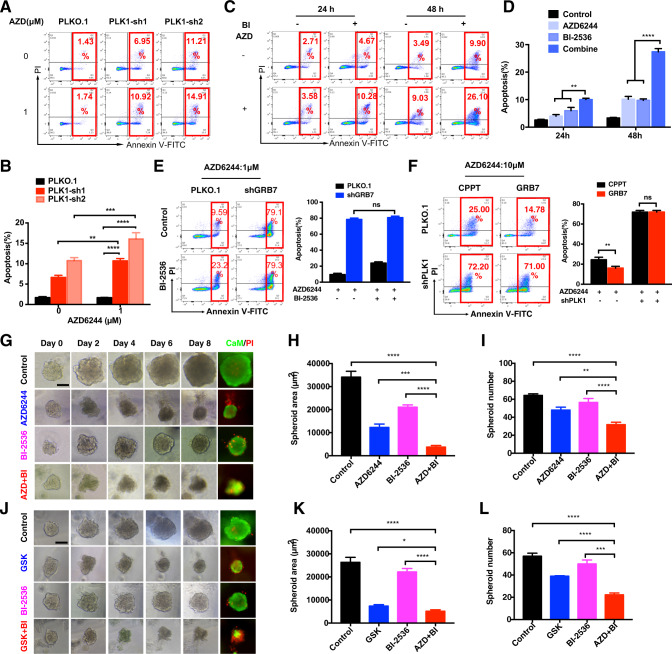
Combined genetic or pharmacologic inhibition of PLK1 and MEKi treatment promoted apoptosis in KRAS mutant colon cancer cells. **A**, **B** Knockdown of PLK1 by shRNAs induced apoptosis upon AZD6244 treatment in HCT116. **B** Quantification of (**A**). Data represented as mean ± SD (*n* = 3). **C**, **D** Apoptosis assay showing that BI-2536 sensitized HCT116 cells to AZD6244. **D** Quantification of (**C**). Data represented as mean ± SD (*n* = 3). **E** HCT116 cells infected with shRNA targeting GRB7 or vector control, then treated with or without BI-2536 in the presence of 1 μM AZD6244 for 48 h. Data represented as mean ± SD (*n* = 3). **F** HCT116 cells infected with vector control, Flag-GRB7, shRNA targeting PLK1, or the combination of both, then treated with 10 μM AZD6244 for 48 h. Apoptosis was detected by annexin V/PI staining. Data represented as mean ± SD (*n* = 3). **G**–**I** Representative phase-contrast and immunofluorescence staining images of HCT116 treated with DMSO, AZD6244, BI-2536, or the combination of both for 8 days in 3D culture system (CaM: green; PI: red). Scale bar, 100 μM. **H** Quantification of spheroid area. **I** Quantification of spheroid number. **J**–**L** Representative phase-contrast and immunofluorescence staining images of SW480 treated with DMSO, GSK, BI-2536, or the combination of both for 8 days in 3D culture system (CaM: green; PI: red). Scale bar, 100 μM. **K** Quantification of spheroid area. **L** Quantification of spheroid number. *p < 0.05, **p < 0.01, ***p < 0.001, or ****p < 0.0001.

We then investigated the synergistic efficacy of combining PLK1 and MEK inhibition in 2D- and 3D-CRC models. The results showed that knockdown of PLK1 alone induced apoptosis, which was further improved with AZD6244 treatment in HCT116 and SW480 cells (Fig. 6A, B, and Supplementary Fig. S7A, B). Similar results were observed in CRC cells with PLK1 knockdown followed by GSK treatment (Supplementary Fig. S7C). In line with the results of GRB7 inhibition, knockdown of PLK1 also significantly promoted apoptosis of A549 and CFPAC-1 cells in the presence of AZD6244 (Supplementary Fig. S7D). To further verify the results of RNAi, we interrupted the function of PLK1 with the small molecular inhibitor, BI-2536. Similarly, combination treatment with BI-2536 and AZD6244 synergistically induced CRC cell apoptosis in HCT116 and SW480 cells after 48 h (Fig. 6C, D, and Supplementary Fig. S7E, F). Next, we further investigated whether GRB7-mediated MEKi resistance was dependent on PLK1. CRC cells were treated with BI-2536 on top of GRB7-knockdown in the presence of AZD6244 (Fig. 6E and Supplementary Fig. S7G). GRB7 knockdown or BI-2536 improved cell apoptosis. However, the combination of these two treatments did not further enhance cell apoptosis, suggesting that GRB7 and PLK1 might not play redundant roles. In addition, apoptosis and clonogenic assays showed that knockdown of PLK1 abolished the drug tolerance derived from GRB7 overexpression in CRC cells (Fig. 6F and Supplementary Fig. S7H-J).

**Fig. 7:**
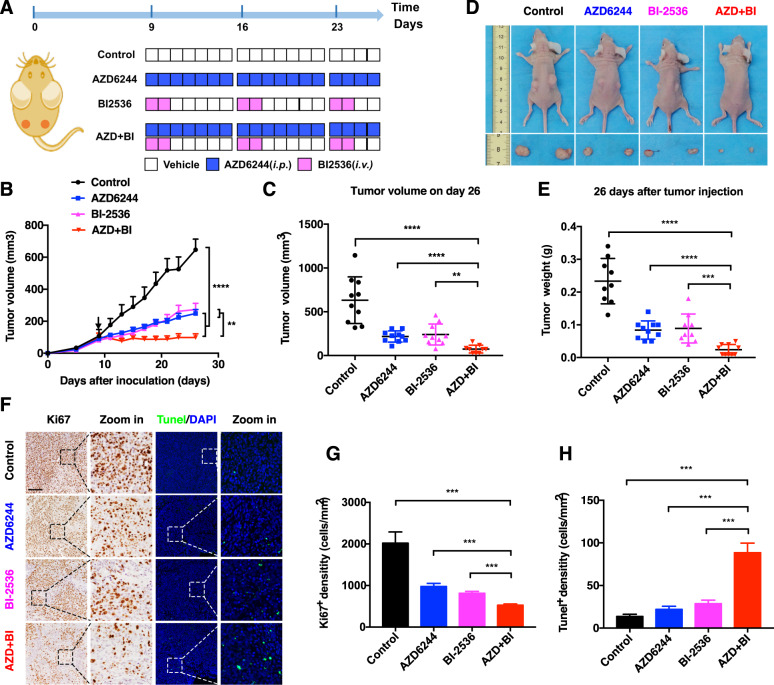
Combination of PLK1i and MEKi synergically suppresses CRC tumor growth. **A** BALB/c Nude mice were subcutaneously inoculated with 3 × 10^6^ HCT116 cells on day 0. Tumor-bearing mice (*n* = 5) were treated with vehicle, AZD6244, BI-2536, or the combination when the tumor volume achieved ≈100 mm^3^. **B** Tumor growth curves for mice treated with vehicle, AZD6244, BI-2536, or the combination. **C** Tumor volume of xenograft tumors on day 26. Data represented as mean ± SD (*n* = 10). **D** Representative image of mice and tumors with different treatments on day 26. (**E**) Weights of xenograft tumors on day 26. Data represented as mean ± SD (*n* = 10). **F**–**H** Representative immunohistochemical images of Ki67 and Tunel staining in tumors from each group. Scale bar, 400 μM. **G** Quantification of Ki67^+^ cells. Data represented as mean ± SD (*n* = 5). **H** Quantification of Tunel^+^ cells. Data represented as mean ± SD (*n* = 5). *p < 0.05, **p < 0.01, ***p < 0.001, or ****p < 0.0001.

Furthermore, synergistic growth inhibition was further validated using a multicellular spheroid three-dimensional (3D) model. HCT116 and SW480 cells were cultured in Matrigel and treated with DMSO, MEKi, PLK1i, or their combination for 8 days. One representative sphere was tracked continually and photographed at the indicated time points. Although MEKi or BI-2536 treatment partially suppressed CRC spheroid growth, BI-2536 treatment significantly improved the therapeutic effects of MEKis. The combination regimen not only significantly inhibited cell proliferation in spheroid numbers and sizes, but also induced apoptosis in HCT116 and SW480 cells (Fig. 6G–L, Supplementary Fig. S8A–F). Encouraged by the in vitro results, we evaluated the combination efficacy in the HCT116 xenograft model (Fig. 7A). Although both AZD6244 and BI-2536 significantly slowed tumor growth, the efficacy of the combination regimen of AZD6244 plus BI-2536 was more potent than that of AZD6244 or BI-2536 alone (Fig. 7B–E). Meanwhile, a clear synergistic effect of the combination treatment was observed on day 26 (Fig. 7C). In line with the tumor growth inhibition, IHC staining showed that the combination AZD6244 and BI-2536 led to a supra-additive reduction in Ki67 protein levels (Fig. 7F, G). Moreover, the number of Tunel-positive cells was significantly increased with the combination treatment (Fig. 7F, H). Taken together, these results demonstrate that the addition of BI-2536 could improve the therapeutic efficiency of MEKi.

**Fig. 8:**
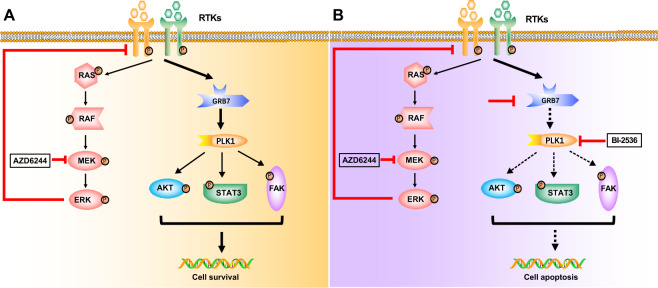
A proposed model depicting the therapeutic mechanism of GRB7-PLK1 axis inhibition to overcome MEKi resistance in KRAS mutant colon cancer cells. **A** In KRAS mutant CRC cells, GRB7 confers MEKi resistance through mediating the RTK pathway. **B** Combination AZD6244 and GRB7-PLK1 axis inhibition significantly suppresses the proliferation and induces apoptosis through attenuating RTK signaling.

Furthermore, similar to the inhibition of PLK1, knockdown of RPTOR also sensitized HCT116 and SW480 cells to AZD6244 (Supplementary Fig. S9A–C). Taken together, our data illustrate the RTK/GRB7/PLK1 signaling pathway (Fig. 8) and highlight the synergistic combination regimen of BI-2536 and MEKi in CRC treatment.

## Discussion

The feedback activations of redundant signaling mediated by RTKs were considered to confer MEKi resistance in previous studies [17, 28, 31, 32]. Recently, RTK-driven feedback activation also conferred resistance to KRAS G12C inhibitors [8], suggesting that the combination of RTK and MAPK-targeted therapy is a promising regimen. Indeed, the heterogeneity of RTK/RTK ligand activation renders combination therapies with MAPKi and RTKi unfeasible [33]. Combination treatment with EGFRi and MEKi produced limited antitumor efficacy in CRC patients [34]. The preexistence or activation of multiple RTKs, except for EGFR-mediated redundant pathways, may be the predominant reason. Therefore, identification of common mediators in the RTK pathway might be critical for the development of novel therapeutic strategies. Herein, we report that GRB7 plays a pivotal role in MEKi resistance in CRC cells with KRAS mutations by performing unbiased genome-wide CRISPR/Cas9 knockout screening, which is a powerful approach to identifying synergistic drug targets [35]. Knockdown of GRB7 induced cell apoptosis and re-sensitized CRC cells to MEKi by attenuating the efficient activation of the RTK pathway. However, ectopic expression of GRB7 was able to activate this signaling pathway, thus protecting CRC cells from MEKi-induced apoptosis and promoting cell survival when the MEK pathway was suppressed. Notably, we observed similar results in KRAS mutant lung and pancreatic cancer cells, implying that GRB7 plays a critical role in RAS-driven cancer cells in the presence of MEKi.

GRB7 targeting inhibits ovarian cancer tumor growth through the ERK inhibition [36]. Interestingly, p-ERK levels were slightly increased in both GRB7 knockdown and GRB7-overexpressing cells (Fig. 4A, B). We speculate that this is due to the DUSP family-mediated compensatory network and further research needs to demonstrate the role of GRB7 in the regulating DUSPs. The inhibition of JNK, which is downstream of GRB7, displays synergy with the MEKi [37, 38].

GRB7 inhibition, either alone, in combination chemotherapy [39, 40], or in combination targeted therapy improves the efficiency of antitumor therapy. However, the detailed regulatory mechanism of GRB7 needs to be illustrated, because effective inhibitors targeting GRB7 are unavailable in the clinic. Here, we found that GRB7 maintained RTK signaling via PLK1 in CRC cells with KRAS mutations. GRB7 overexpression promoted the cytoplasmic localization of PLK1, which might be attributed to the nuclear export complex consisting of GRB7 and its function as a translational regulator [30]. Similar to the knockdown of GRB7, inhibition of PLK1 with shRNAs and inhibitor, attenuated the GRB7 oncogenic signals, leading to cell apoptosis in the presence of MEKi. In addition, extra BI-2536 did not further enhance cell apoptosis induced by GRB7 knockdown in the presence of MEKi. PLK1 signaling has also been reported to confer resistance to PI3K inhibitors by Tie-FGFR1-STAT3 [41]. However, we observed that inhibition of PLK1 with shRNAs or inhibitor significantly impaired the phosphorylation of STAT3, which was induced by AZD6244.

PLK1 was identified as a lethal kinase in RAS-mutated CRC, and the potent efficacy of PLK1 inhibitor has been well demonstrated in vitro and in vivo [42]. Recently, the PLK1 inhibitor onvansertib was fast tracked to treat patients with KRAS mutations in CRC by the FDA. Our study found that the PLK1 and MAPK pathway synergistically controlled cell proliferation and apoptosis. The combination of PLK1 and MEKis showed more potent efficacy in suppressing cell proliferation in 2D and 3D cellular models, as well as in xenograft animal model. We also noticed that an additional MEKi significantly improved the efficiency of the PLK1 inhibitor by diminishing the dose from 50 [42] to 10 mg/kg (Fig. 7A–C). Furthermore, we showed that RPTOR, PLK1, and GRB7 formed a complex, and their knockdown synergistically inhibited CRC cell proliferation with MEKi, consistent with the fact that activation of the mTOR pathway plays a pivotal role in RTKi resistance [43]. Recently, PLK3, another member of the PLK1 family, was reported to confer resistance to the MAPK pathway inhibitor in melanoma [44]. Thus, the detailed function of the PLK1 family in the regulation of MEKi resistance in CRCs needs to be further investigated.

In conclusion, our data illustrate that the RTKs-GRB7-PLK1 signaling pathway plays a pivotal role in resistance to MEKis and a combinational inhibition of this axis and MEK provides a promising strategy to cure CRC patients with KRAS mutation (Fig. 8). Moreover, lung and pancreatic cancer cells with KRAS mutation were also sensitive to GRB7-PLK1 inhibition in combination with MEKi. This provides inspiration for novel treatment combinations for refractory tumors with KRAS mutation.

## Materials and methods

### Cell culture

HT29, WIDR, HCT116, LS174T, and HEK293T; SW480 and A549; and CFPAC-1 cells were cultured in DMEM; RPMI; and IMDM, respectively, in a humidified 5% CO_2_ incubator at 37 °C. The culture media were supplemented with 10% fetal bovine serum (FBS) and 1% penicillin/streptomycin. All cell lines were purchased from ATCC and verified to be free of mycoplasma.

### Cell apoptosis

Cell apoptosis assays were performed as previously described [45]. Cells were stained using the Annexin V Apoptosis Detection Kit (4A Biotech) and analyzed using CytoFLEX (Beckman Coulter). The early apoptotic cell population (Annexin V^+^/PI^−^) and late apoptotic cell population (Annexin V^+^/PI^+^) were combined to obtain the total proportion of apoptotic cells.

## Supplementary information

Supplementary information

Supplementary Table S1

Supplementary Table S2

Supplementary Table S3

## Data Availability

All data generated or analyzed during this study are included in this published article and its supplementary information files.
