# Supplementary material for: Genome-wide CRISPR-cas9 knockout screening identifies GRB7 as a driver for MEK inhibitor resistance in KRAS mutant colon cancer
Source: Oncogene. 2021 Oct 30;41(2):191–203. doi: 10.1038/s41388-021-02077-w (PMC8732282; doi:10.1038/s41388-021-02077-w)
Supplement: Supplementary file 1 — Supplementary information [file 41388_2021_2077_MOESM1_ESM.docx]

**Supplementary information**

Supplementary Table S1. CRISPR-MEKi screening and transcriptome data in HCT116 cells

Supplementary Table S2. Bliss Index of combination treatment

Supplementary Table S3. Mass spectrometry of GRB7 immunoprecipitation in HCT116 cells

Supplementary Figure S1. CRISPR-Cas9 knockout library screening in CRC cells

Supplementary Figure S2. GRB7 was upregulated in colon cancer, pancreatic cancer, and lung cancer tissues rather than in melanoma tissues

Supplementary Figure S3. Combinatorial inhibition of GRB7 and MEK decreased cell proliferation and induced cell apoptosis

Supplementary Figure S4. GRB7 overexpression promoted resistance to MEKi in KRAS mutation colon cancer cells through RTK pathway activation

Supplementary Figure S5. GRB7 binds with PLK1

Supplementary Figure S6. Combinatorial inhibition of PLK1 and MEK impaired the activation of RTK pathway

Supplementary Figure S7. Combination inhibition of PLK1 and MEK promoted cell apoptosis

Supplementary Figure S8 Combination inhibition of PLK1 and MEK suppressed cell growth in 3D colorectal cancer cell culture system

Supplementary Figure S9. Combination inhibition of RPTOR and MEK promoted cell apoptosis

**Supplementary Materials and Methods**

**Reagents and Antibodies**

AZD6244 (S100), puromycin (S7417), and BI-2536 (S1109) were purchased from Selleck. GSK1120212 (GSK) was a gift from Novartis. Stocks and dilutions of the drugs were prepared in dimethyl sulfoxide (DMSO, Sigma). Blasticidin (ant-bl-1) was purchased from Invitrogen. Antibodies against phospho-MAPK (ERK1/2) (4370), MAPK (ERK1/2) (4695), KI67 (9027), AKT (4691), p-AKT (4060), p-STAT3 (4113), STAT3 (49139), PARP (9532), p-FAK (8556), FAK (3285), p-mTOR (5536), m-TOR (2983), p-4EBP1 (2855), 4EBP1 (9644), RPTOR (2280), and PLK1 (9062) were purchased from Cell Signaling Technology (CST); antibodies against PLK1 (ab17056) and GRB7 (ab183737) were purchased from Abcam; antibody against FLAG (F1804) was purchased from Sigma, antibodies against β-ACTIN (TA-09) were purchased from ZsBio.

**CRISPR/Cas9 screening and data analysis**

The Human GeCKOv2 CRISPR knockout pooled library was purchased from Addgene (#1000000049). Stable Cas9 expressing cells were established by lentiviral transduction followed by blasticidin selection. The cells were then infected with the lentiviral GeCKOv2 library pool at an MOI of 0.3 for coverage of approximately 500 times. The transduced cells were selected with 2 µg/mL of puromycin for 5 days to generate a mutant cell pool, which was then treated with AZD6244(1 μM) or solvent (DMSO) for 7 days. After treatment, the cells were collected for sequencing. CRISPR-sgRNA sequencing was performed as previously described[1, 2]. Briefly, genomic DNA from the cells was obtained by phenol/chloroform extraction. The sgRNA sequences were amplified using NEB Next® High-Fidelity 2X PCR Master Mix, and NGS sequencing was performed on the Illumina HiSeq platform.

The sequencing reads were aligned to the sgRNA library file to obtain the read counts per guide using MAGeCK (v0.5.6). Considering the off-target effects of sgRNA, we selected genes with at least three sgRNAs that were down-regulated in AZD6244-treated cells as preliminary candidates. In parallel, we profiled the transcriptome of HCT116 cells treated with or without AZD6244 for 7 days. We assumed that the resistance-promoting genes were effectively transcripted in either DMSO- or AZD6244-treated cells. Thus, the preliminary genes with a TPM value of no less than 10 (moderate expression level) and highly expressed in AZD6244-treated cells were identified as candidate genes[3]. Based on this strategy, we identified a series of sgRNAs targeting 1846 genes that were significantly depleted in AZD6244-treated cells compared with those in DMSO-treated cells. To explore the function of the significantly down-regulated genes, the Enrichr (v3.0) package was used for enrichment analysis, based on Fisher’s exact test and the Benjamini & Hochberg (BH) method. First, the latest updated gene ontology biological process annotations (GO_Biological_Process_2021) were obtained by EnrichR, and enrichment of downregulated genes was performed by Fisher's exact test using the Enrichr package. Next, pathways with an adjusted *p* value < 0.05 (BH method) were considered as significantly enriched. Finally, the most significantly enriched signaling pathways in HCT116 cells treated with AZD6244 were selected according to the number of genes enriched and displayed in the bubble chart.

**Immunofluorescence**

Slides were prepared in 12-well plates and cells were seeded on the slides overnight. The cells were fixed with 4% paraformaldehyde for 20 min at room temperature after inhibitor treatment. After fixation, 0.1% Triton X-100 was used for permeabilization. The permeabilized cells were blocked with 1% BSA for 1 h at room temperature. Cells were then incubated with primary antibodies against GRB7 and PLK1 overnight at 4°C. Bound primary antibodies were detected by incubation with Alexa Fluor 488- or 594-conjugated secondary antibodies (Invitrogen) for 1 h at room temperature. Fluorescence images were acquired using confocal microscope (Nikon).

**Lentiviral cDNA/shRNA constructs and virus infection**

cDNA and shRNA were cloned into the pRRLsin.cPPT.CMV.IRES lentiviral expression vector (CPPT) or pLKO.1 puro vector, respectively. cDNA or shRNA lentivirus were packaged in HEK293T cells and transduced into targeted cells as previously described[4]. The sequence of shRNA used were as following: GRB7-sh1 (CGCCAAGTACGAACTGTTCAA); GRB7-sh2 (CCAGGGCTTTGTCCTCTCTTT); GRB7-sh3 (GCCATCTGCATCCATCTTGTT); GRB7-sh4 (CCTTGAGAAGTGCCTCAGATA); PLK1-sh1 (CCCGAGGTGCTGAGCAAGAAA); PLK1-sh2 (CGATACTACCTACGGCAAATT); RPTOR-shRNA (CGTATCATCCTTAAACGTTAT).

**RNA extraction, sequencing, and data analysis**

Total RNA was isolated from CRC cells with or without AZD6244 using TRIzol (Invitrogen) as per the manufacturer's recommendations. RNA-seq libraries were generated with the TruSeq RNA Library Prep Kit according to the manufacturer’s protocol. Enriched RNA-seq libraries were multiplexed and sequenced on an Illumina HiSeq 4000 platform. Sequencing reads were aligned to the human reference genome GRCh38(ftp://ftp.ensembl.org) and quantified by Salmon v0.81.

**Immunoblot and IP analysis**

For western blot analysis, cell lysates or immunoprecipitates were separated by 10 – 12% SDS-PAGE gels and then transferred to PVDF membranes (Millipore). The membranes were blocked with 5% BSA for 1 h. They were then incubated with the indicated primary and secondary antibodies. Western lightning ECL reagent (Millipore) was used for signal detection. For IP, cell lysates were incubated with 2 μg of primary antibodies or IgG control at room temperature for 2 h, followed by incubation with protein G-agarose beads at 4°C overnight. Beads were then washed five times and subjected to western blot analysis.

**Cell viability assay**

Cells were seeded in flat-bottomed 96-well plates at 2 000 cells/well and treated with AZD6244 on the following day. Cells were then incubated for another 72 h, and cell viability was measured by MTS (Promega, #G3582) as per the manufacturer's recommendations. Relative survival in the presence of AZD6244 was normalized to that of DMSO after background subtraction. Survival curve analysis was performed by GraphPad Prism 7.0 software.

**Clonogenic assays**

Cells were seeded into 6-well plates (1 × 10^4^ cells per well) and allowed to adhere overnight. On the following day, the cells were cultured with or without inhibitors, as indicated. Drugs or medium were replenished every 2 days. Colonies were fixed in 4% paraformaldehyde and stained with 0.05% crystal violet for 10-14 days. Quantitative analysis was performed by ImageJ software.

**Bliss Analysis**

The potential synergistic effects of GRB7/PLK1 inhibition and MEKi were determined by Bliss analysis as[5]: Y_ab,P_ = Y_a_ + Y_b_ – Y_a_Y_b_, where Y_a_ equals the percentage inhibition (apoptosis) by the treatment a and Y_b_ the percentage inhibition (apoptosis) of treatment b. Synergistic effects were defined as the percentage of observed effects greater than Y_ab,P_.

***In vivo* studies**

Female BABL/c Nude mice were obtained from Beijing HFK Bioscience

Co. Ltd., Beijing, China). All animal experiments were performed according to the protocols approved by the Ethics Review Committee of Animal Experimentation of Sichuan University. Six weeks old mice were injected with 3 x 10^6^ HCT116 cells at two sites on the flanks of each mouse. Tumor volumes were measured with calipers in two dimensions (length and width) every two days. Tumor size was calculated using the formula: (length × width^2^)/2. Body and tumor weight were measured using a balance. Mice that developed tumors reaching 80–100 mm^3^ in size were randomized into four groups with five mice in each group: 1) solvent, 2) AZD6244, 3) BI2536, and 4) AZD6244 plus BI2536. AZD6244, solubilized in a methocel/polysorbate buffer, was injected daily by intraperitoneal injection at a dose of 12.5 mg/kg. BI2536, solubilized in a methocel/polysorbate buffer, was injected twice a week by intravenous injection at a dose of 10 mg/kg. Mice were randomized for treatment and all animal experiments were performed by well-trained researchers blind to the treatments.

**Bioinformatics analysis for GEO RNA-seq datasets**

GSE108050, GSE98922, and GSE114061 datasets were used to profile the effects of MEKi treatment on the RTK pathway. Heatmaps of the RTK genes were generated using a pheatmap (v1.0.12). For gene set enrichment analysis (GSEA), the samples were first divided into MEKi-treated and DMSO-treated groups according to the culture conditions. Second, the log2 fold change (log2FC) was calculated by comparing the MEKi-treated cells to the DMSO-treated cells using the DESeq2 package (v1.26.0). The genes were ranked according to the log2FC in each dataset. Third, the robust rank aggregation (RRA) algorithm of the RobustRankAggreg package (v1.1) was used to aggregate the above gene lists to obtain a comprehensive gene ranking. Finally, the comprehensive ranking was used for GSEA analysis using the clusterProfiler package (v3.14.3). *The p* value and adjusted *p* value were determined by the permutation test and the Benjamini & Hochberg (BH) method, respectively. If the adjusted *p-value* was less than 0.05, the pathway was considered to be significantly enriched. The members of the gene signature were ranked according to the GSEA results, and those that were distributed at the top of the sorted gene list (the left side of the panel) or had an NES greater than 1, were considered to be positively correlated with the treatment using MEKi.

**Bioinformatics analysis of The Cancer Genome Atlas (TCGA) RNA-seq datasets**

We downloaded clinical, gene expression, and somatic mutation data from TCGA data portal (https://portal.gdc.cancer.gov/) for [colon cancer (COAD)](https://xenabrowser.net/datapages/?cohort=GDC%20TCGA%20Colon%20Cancer%20(COAD)&removeHub=https://xena.treehouse.gi.ucsc.edu:443), [melanoma (SKCM)](https://xenabrowser.net/datapages/?cohort=GDC%20TCGA%20Melanoma%20(SKCM)&removeHub=https://xena.treehouse.gi.ucsc.edu:443), pancreatic cancer (PAAD), [lung adenocarcinoma (LUAD),](https://xenabrowser.net/datapages/?cohort=GDC%20TCGA%20Lung%20Adenocarcinoma%20(LUAD)&removeHub=https://xena.treehouse.gi.ucsc.edu:443) and [lung squamous cell carcinoma (LUSC)](https://xenabrowser.net/datapages/?cohort=GDC%20TCGA%20Lung%20Squamous%20Cell%20Carcinoma%20(LUSC)&removeHub=https://xena.treehouse.gi.ucsc.edu:443). For the statistical analysis, LUAD and LUSC were combined together and collectively called lung cancer. COAD, SKCM, PAAD, and lung cancer patients were classified into different mutant subtypes based on mutation information.

COAD patients were divided into high and low groups according to the median cutoff of the GRB7 expression level, and Kaplan-Meier analysis was performed using survival (v3.2-7) and survminer (v0.4.8) packages. The log-rank test was used to determine *p* values.

**Mass spectrometry (MS)-based proteomics**

HCT116 cells expressing FLAG-tagged GRB7 were lysed with RIPA buffer and immunoprecipitated using an anti-FLAG antibody as described above. The immunoprecipitates were washed twice with 50 mM NH_4_HCO_3_. Proteins were reduced (10 mM dithiothreitol, 37 °C for 4 h) and alkylated (50 mM iodoacetamide, room temperature in the dark for 40 min), and subsequently digested with trypsin at an enzyme/substrate ratio of 1:50 at 37 °C overnight. Peptides were desalted using C18 SpinTips (Protea), loaded on a pre-column, and separated by reverse phase HPLC (Thermo Easy nLC1000) over a 140-min gradient before nano-electrospray mass spectrometry using a Q Exactive mass spectrometer (Thermo). The parameters for the full-scan MS were as follows: resolution of 70,000 across 350–1800 m/z, AGC 3e6, and maximum IT 40 ms. Raw mass spectral data files (.raw) were searched using Proteome Discoverer (Thermo) and Mascot version 2.4.1 (Matrix Science). The Mascot search parameters were as follows: 20 ppm mass tolerance for precursor ions; 0.6 Da for fragment mass tolerance; 2 maximum missed cleavages of trypsin; fixed modification with carbamidomethyl (cysteine); and variable modification with methionine oxidation.

**Spheroid formation assay**

To create non-adhesive conditions, a 6-well plate was coated with 1 mL of 0.6% agarose dissolved in DMEM. After agarose polymerization, 2 x 10^5^ cells per well (in 2 mL of medium) were seeded on top of the agarose overnight. Spheroids were then harvested and embedded in Matrigel (BD 356234) in a 96-well plate. On the following day, the spheres were cultured with or without inhibitors, as indicated. Drugs or media were replenished every 2 days. After 8 days, the spheres were washed three times with PBS and stained with FITC-Calcein-AM (CaM) and PI. Images of the spheroids were captured with a fluorescence microscope (Nikon).

**Quantitative Real-time PCR**

Total RNA was extracted from HCT116 cells using TRIzol reagent (Invitrogen). cDNA was synthesized from 4 μg of total RNA using the Revert Aid First Strand cDNA Synthesis Kit (Thermo Fisher Scientific). Quantitative PCR was performed using the Universal SYBR® Green Super mix (Bio-Rad). Actin was used as a reference gene for the relative quantification. The primers used for qPCR were as follows: human ACTIN (forward: CATGTACGTTGCTATCCAGGC; reverse: CTCCTTAATGTCACGCACGAT), human GRB7 (forward: GTGCCTCAGATAATACCCTG; reverse: GGTTTGTCTTCTTCCTCCAG), human PLK1 (forward: TGCTCTTCAATGACTCAACA; reverse: TTCATCAAGGAGTTGGGATG); human RPTOR (forward: CAAGTACTTCCTGTCGGT; reverse: CCGTGTGATAGCTGTTGA).

**Immunohistochemistry (IHC) staining and Tunel assay**

To prepare the tumor samples for IHC staining, the tumor pieces were fixed with 10% formalin, followed by paraffin embedding. Tumor sections of 4 μm thickness were mounted on glass slides for IHC staining, as described previously[6]. For GRB7, PLK1, and KI67 immunohistochemistry staining, the slides were deparaffinized, incubated in 3% hydrogen peroxide, and antigen retrieval was performed in EDTA (pH = 9.0) for 8 min in a pressure cooker. The slides were incubated with primary antibodies overnight, followed by incubation with appropriate horseradish peroxidase-conjugated secondary antibodies at room temperature for 60 min. Finally, the slides were incubated with 3,3’-diaminobenzidine (DAB) for visualization. The IHC score was determined according to the percentage of positively stained cells (0, negative staining; 1, < 20% positively stained cells; 2, 20%–50% positively stained cells; 3, > 50% positively stained cells). Apoptosis of the cancer cells was determined by staining the slides with the DeadEnd^TM^ Fluorometric Tunel Kit (Promega, #G3250) according to the manufacturer’s instructions.

**Statistical analysis**

Data are presented as mean ± SEM unless otherwise stated. The sample size was determined based on prior experiments. The significance attached to the comparison between among groups was determined with GraphPad Prism 7 software using the Student’s *t*-test, ANOVA, or the Wilcoxon test according to the specific circumstance, respectively. where ∗*p* < 0.05, ∗∗*p* < 0.01, ∗∗∗*p* < 0.001, or ∗∗∗∗*p* < 0.0001. F test was used to compare variances within each group of data. Pearson’s R was used to calculate the correlation between two continuous variables. The log-rank test was used to compare the survival distribution of patients in different groups.

**Supplementary figures**

**Figure S1**


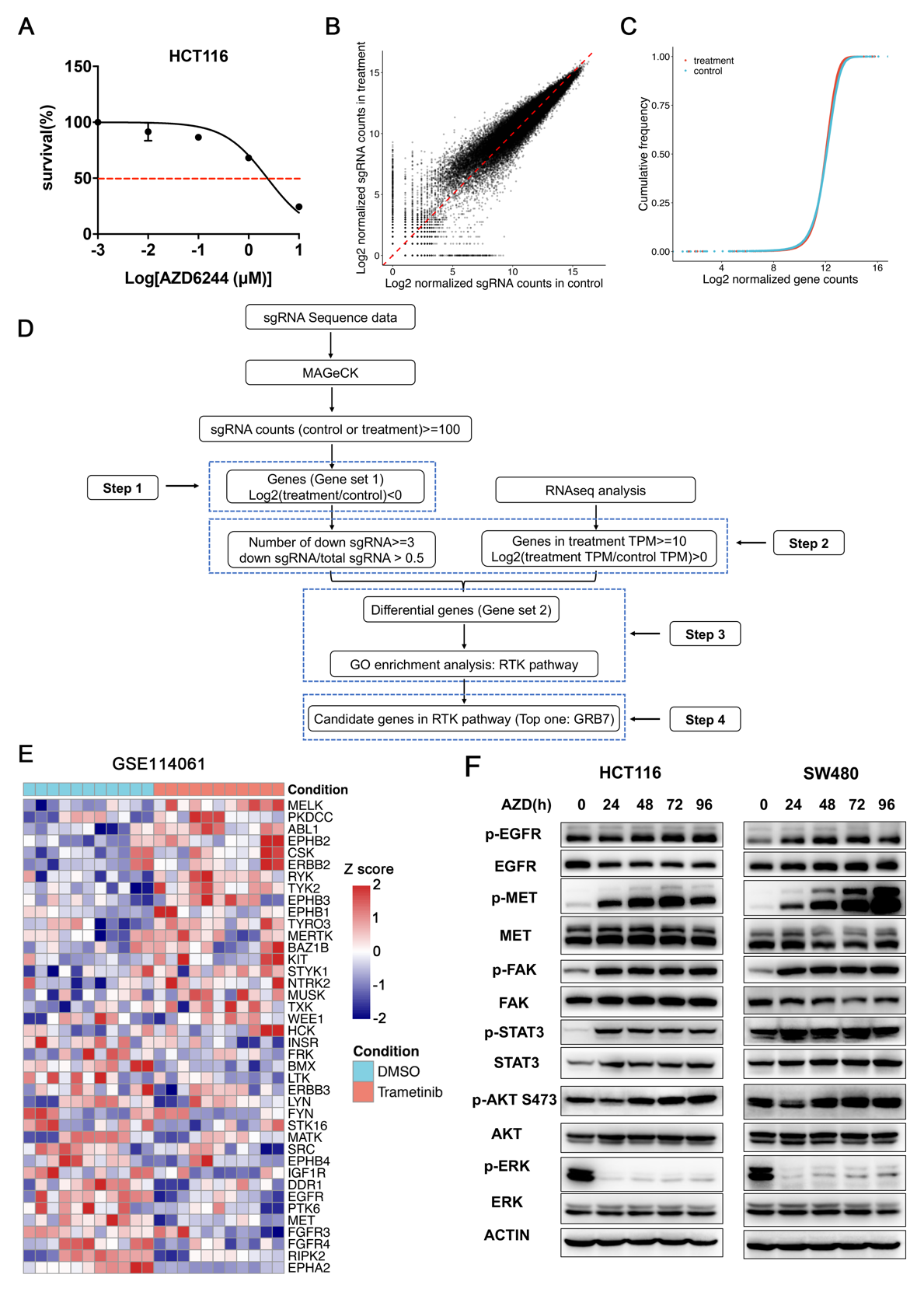


**Figure S1 CRISPR-Cas9 knockout library screening in CRC cells**

**(A)** Survival curves of HCT116 treated with the AZD6244 for 72 hours. Results are shown relative to DMSO-treated controls (mean ± SD, n = 5; dashed line, 50% inhibition). **(B)** Scatter plot of sgRNA representation (log2 number of reads) between with or without drug treatment in HCT116 cells. **(C)** Cumulative frequency of sgRNAs in HCT116 cells treated with or without AZD6244. **(D)** Analysis pipeline of genome wide CRISPR/Cas9 knockout library screening. **(E)** Heatmap of RTKs genes in CRC organoids treated with MEKi compared with DMSO control in GEO dataset. **(F)** Western blot analysis of RTK pathway proteins in HCT116 and SW480 cells treated with AZD6244 at indicated times.

**Figure S2**


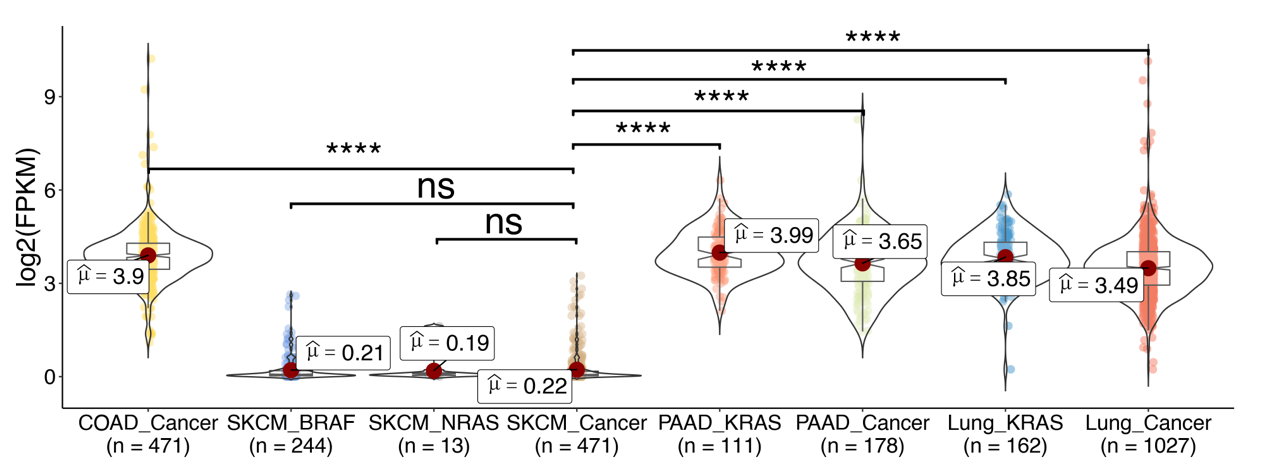


**Figure S2 GRB7 was upregulated in colon cancer, pancreatic cancer, and lung cancer tissues rather than in melanoma tissues**

Boxplot of GRB7 expression in tumor tissues from colon cancer (COAD), melanoma (SKCM), pancreatic cancer (PAAD), and lung cancer patient samples from TCGA datasets. The *p* value was determined by Wilcoxon test. ∗∗∗∗*p* < 0.0001, ns (not significant).

**Figure S3**

**
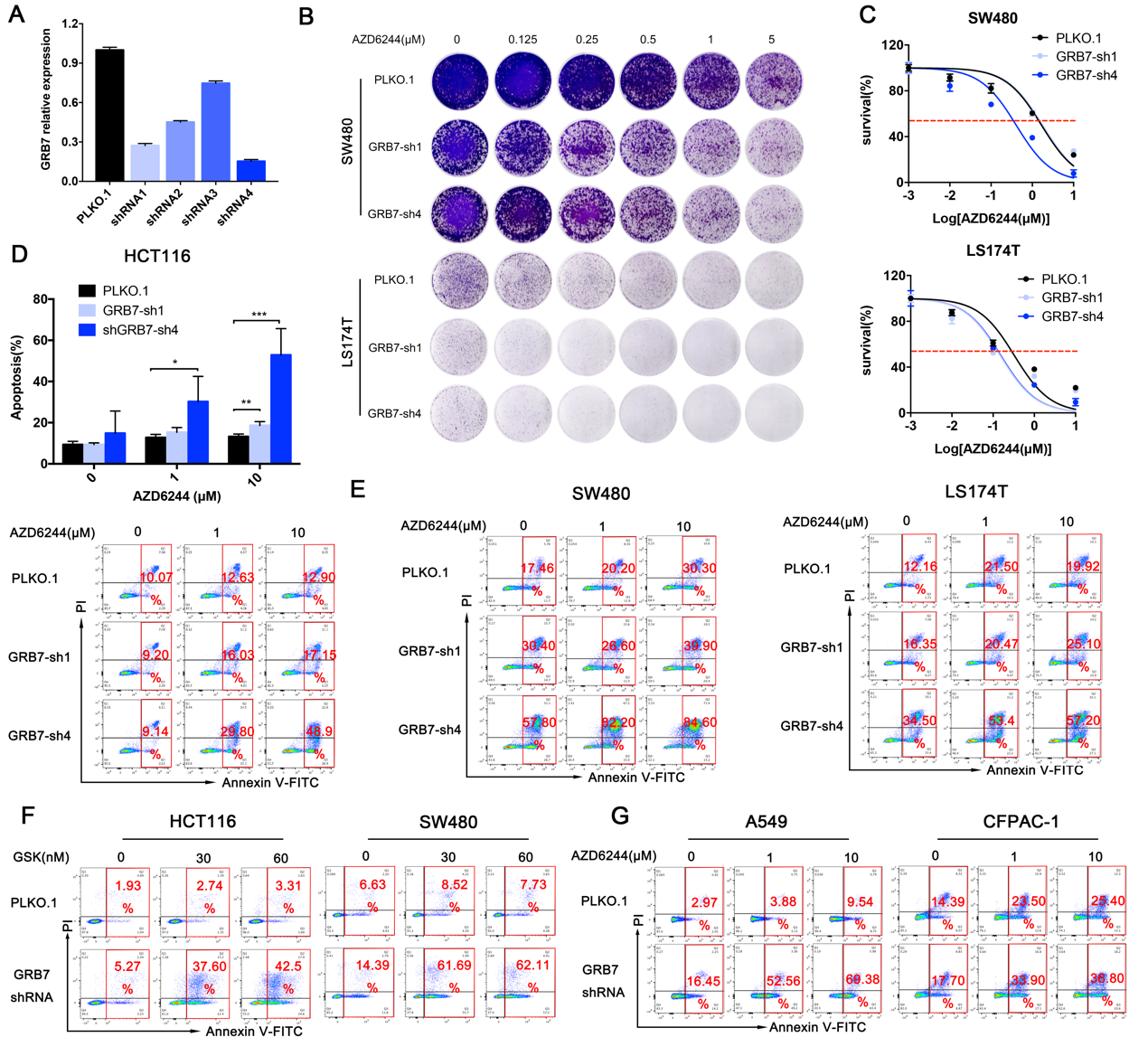
**

**Figure S3 Combinatorial inhibition of GRB7 and MEK decreased cell proliferation and induced cell apoptosis**

**(A)** qPCR analysis identified that the efficiency of shRNAs targeted GRB7. Mean ± SD of 3 replicates is shown. **(B)** Long-term colony assays of SW480 and LS174T infected with shRNAs targeted with GRB7 or vector control treated with AZD6244. **(C)** Survival curves of SW480 and LS174T infected with shRNAs targeted with GRB7 or vector control titrated with the AZD6244 for 72 hours. Results are shown relative to DMSO-treated controls (mean ± SD, n = 5; dashed line, 50% inhibition). **(D)** Apoptosis assay of HCT116 infected with shRNAs targeted with GRB7 or vector control treated with AZD6244 for 24h. Data represented as mean ± SD (n = 3). **(E)** Apoptosis assay of SW480 and LS174T infected with shRNAs targeted with GRB7 or vector control treated with AZD6244 for 48h. **(F)** Apoptosis assay of HCT116 and SW480 infected with shRNAs targeted with GRB7 or vector control treated with GSK for 24h. **(G)** Apoptosis assay of A549 and CFPAC-1 infected with shRNAs targeted with GRB7 or vector control treated with AZD6244 for 24h. ∗*p* < 0.05, ∗∗*p* < 0.01, ∗∗∗*p* < 0.001, or ∗∗∗∗*p* < 0.0001.

**Figure S4**


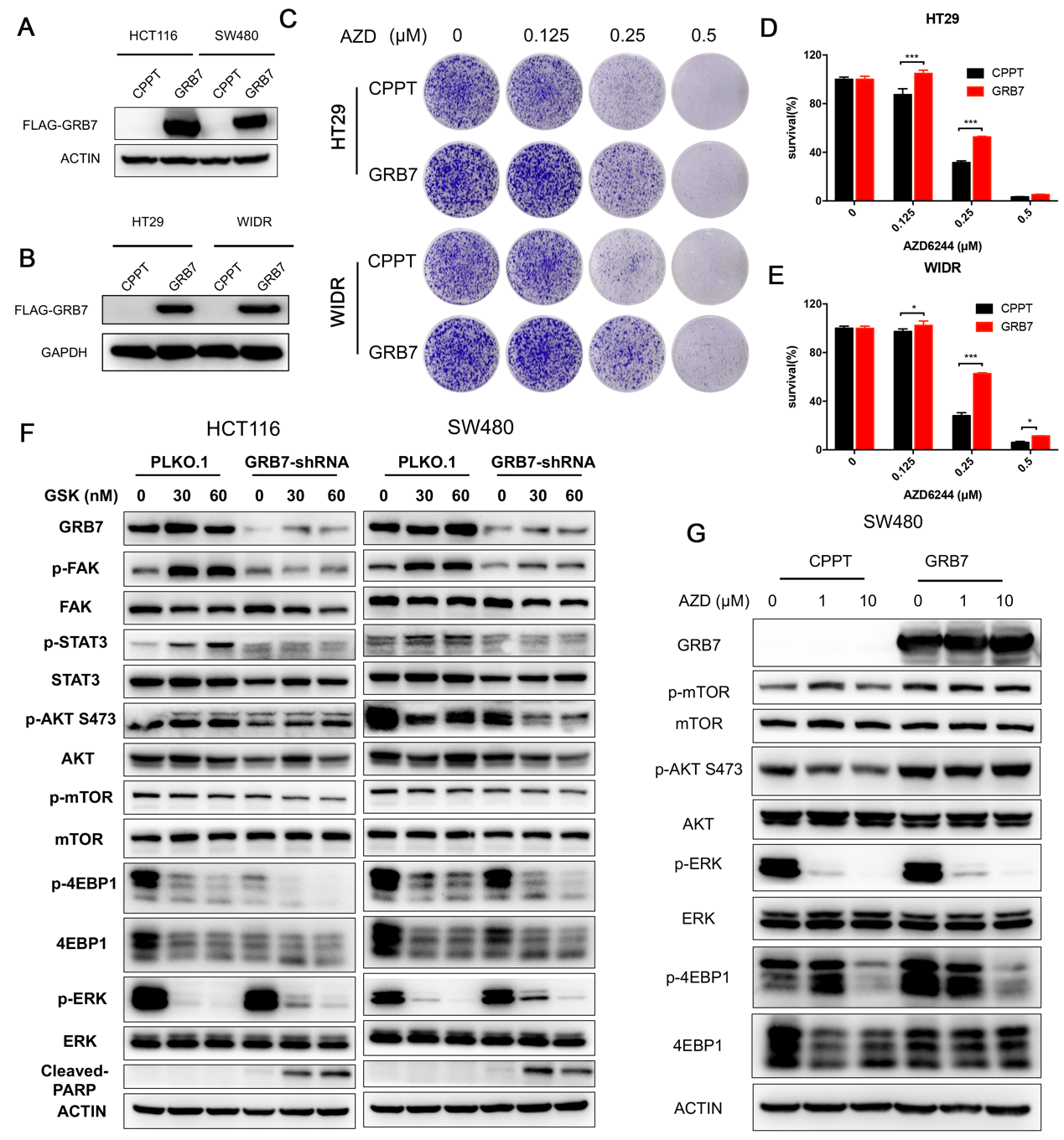


**Figure S4 GRB7 overexpression promoted resistance to MEKi in KRAS mutation colon cancer cells through RTK pathway activation**

**(A and B)** HCT116 **(A)**, SW480 **(A)**, HT29 **(B)** and WIDR **(B)** cells were ectopically expressed with GRB7 or vector control. Then cell lysates were made for immunoblot for indicated proteins. **(C-E)** Long-term colony assays of HT29 and WIDR expressing GRB7, or vector control treated with AZD6244. **(D)** Quantification of HT29, **(E)** Quantification of WIDR. Data represent mean ± SD (n = 3). **(F)** HCT116 and SW480 cells were infected with shRNAs targeted with GRB7 or vector control, then treated with GSK for 24h. Cells lysates were made for immunoblot analysis with indicated antibodies. ACTIN was used as loading control. **(G)** SW480 were ectopically expressed with GRB7 or vector control. After infection, cells were treated with AZD6244 at indicated concentration for 24h. Cell lysates were made for immunoblot analysis with indicated antibodies. ACTIN was used as loading control. ∗*p* < 0.05, ∗∗*p* < 0.01, ∗∗∗*p* < 0.001, or ∗∗∗∗*p* < 0.0001.

**Figure S5**


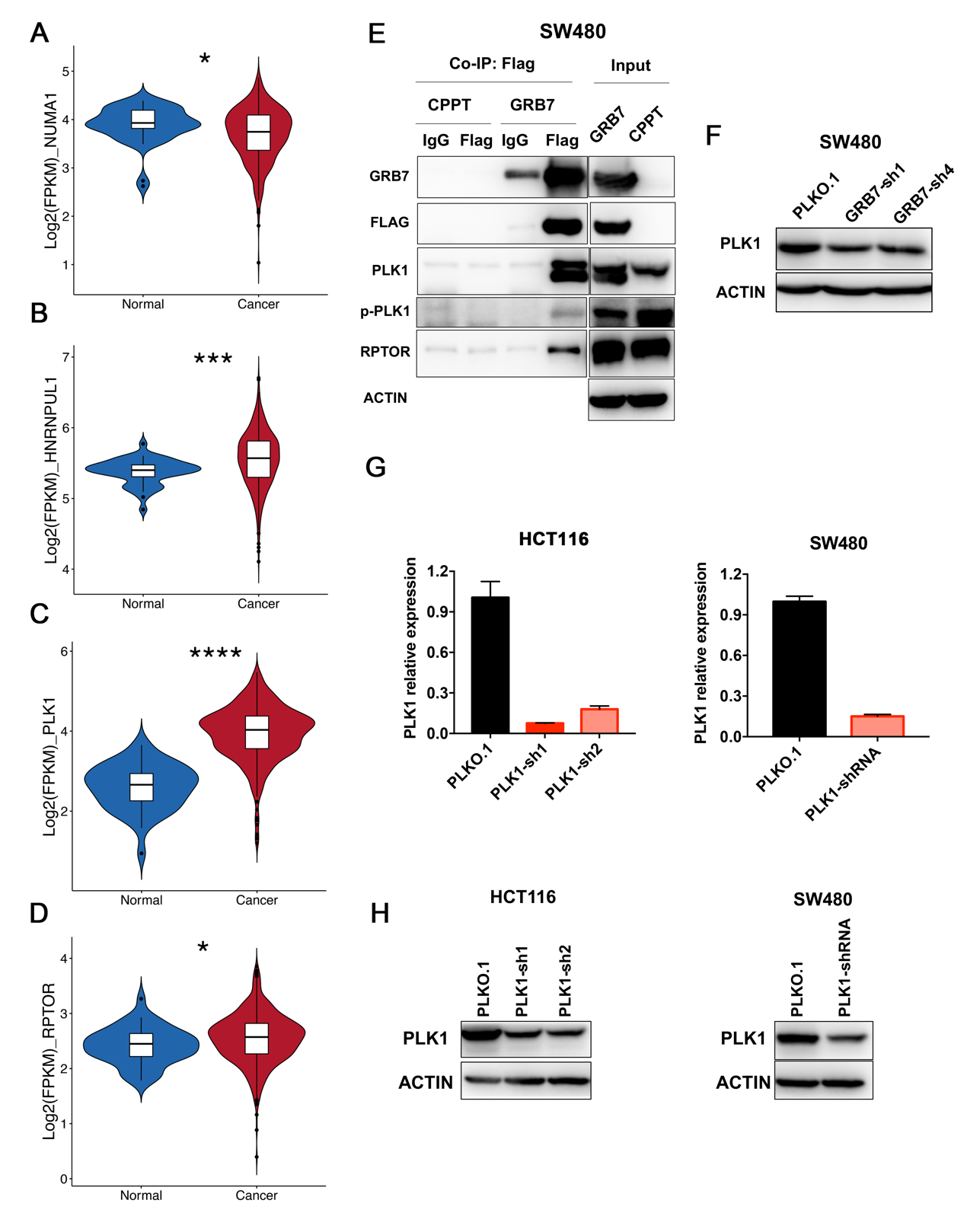


**Figure S5 GRB7 binds with PLK1**

**(A-D**) mRNA expression of NUMA **(A)**, HNRNPUL1 **(B)**, PLK1 **(C)** or RPTOR **(D)** in normal and tumor tissues from colon cancer patients (Data from TCGA cohort). The *p* value was determined by Wilcoxon test. **(E)** Cell lysates from GRB7 expressing or vector control cells were subjected to immunoprecipitation with Flag antibody or immunoglobulin G control. The immunoprecipitants or cell lysates were then blotted with the indicated antibodies. **(F)** SW480 were transfected with vector (control) or shRNA targeting GRB7 and treated with AZD6244 for 24h. The cell lysates were made for western blot analysis of indicated proteins. **(G)** qPCR analysis of PLK1 in HCT116 and SW480 infected with shRNAs targeting PLK1 or vector control. Data represented as mean ± SD (n = 3). **(H)** HCT116 infected with shRNA targeting PLK1 or vector control, then the lysates were made for immunoblot analysis. ∗*p* < 0.05, ∗∗*p* < 0.01, ∗∗∗*p* < 0.001, or ∗∗∗∗*p* < 0.0001.

**Figure S6**

**
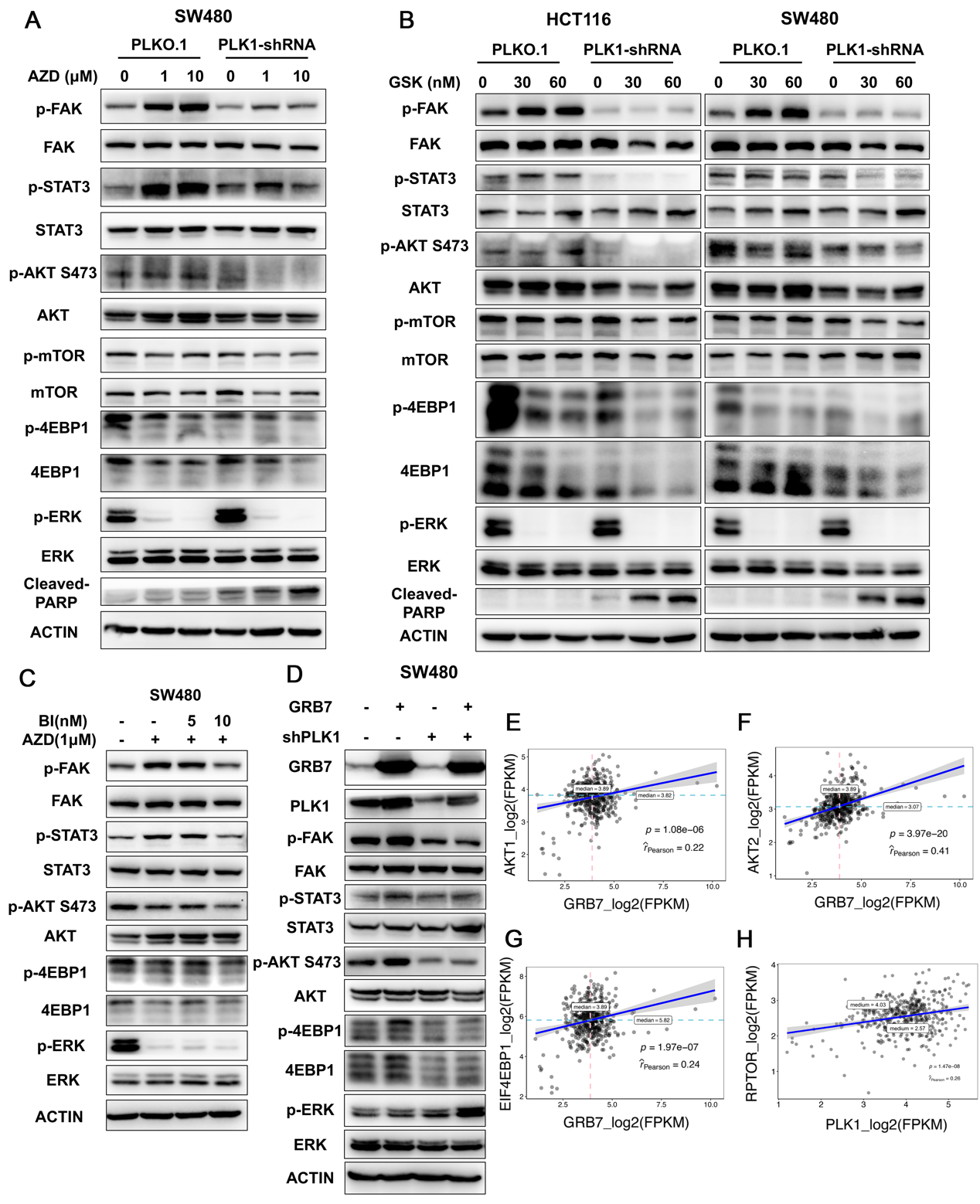
**

**Figure S6 Combinatorial inhibition of PLK1 and MEK impaired the activation of RTK pathway**

**(A)** HCT116 were transfected with vector (control) or shRNA targeting PLK1, then treated with AZD6244 for 24h. Cell lysates were made for immunoblot analysis. **(B)** HCT116 and SW480 cells were infected with shRNAs targeted with PLK1 or vector control, then treated with GSK for 24h. Cells lysates were made for immunoblot analysis with indicated antibodies. ACTIN was used as loading control. **(C)** HCT116 were treated with DMSO, AZD6244, or AZD6244 plus BI2536 for 24h. Cell lysates were made for immunoblot analysis. **(D)** the western blot analysis of overexpression of GRB7 and knockdown of PLK1 in SW480 cells. **(E-G)** Correlation analysis of GRB7 with AKT1 **(E)**, AKT2 **(F)**, and EIF4EBP1 **(G). (H)** Correlation analysis of PLK1 with RPTOR in tumor tissues from colon cancer patients (Data from TCGA cohort). The *p* value was determined by Student's *t*-test. ∗*p* < 0.05, ∗∗*p* < 0.01, ∗∗∗*p* < 0.001, or ∗∗∗∗*p* < 0.0001.

**Figure S7**


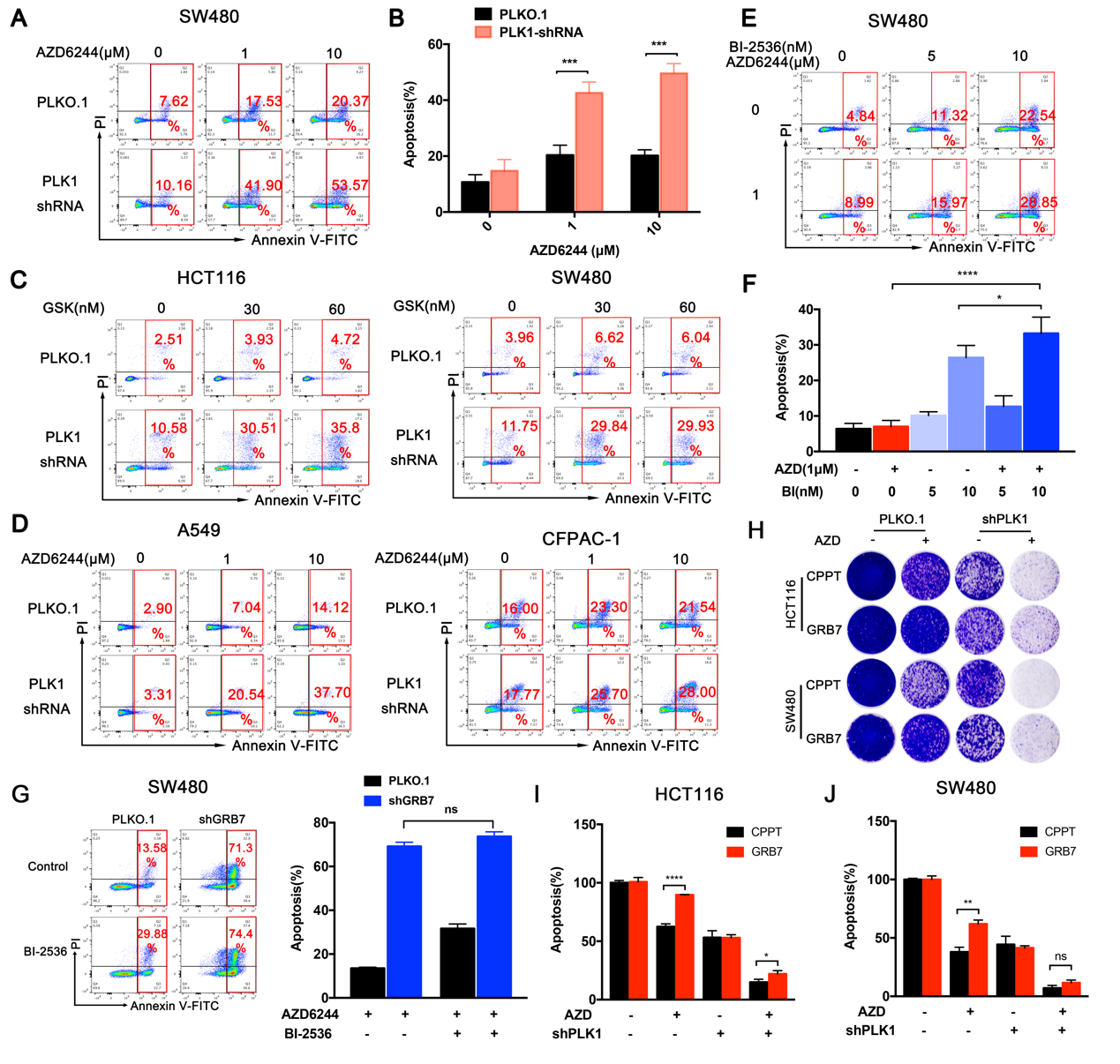


**Figure S7 Combination inhibition of PLK1 and MEK promoted cell apoptosis**

**(A and B)** SW480 were infected with shRNA targeted with PLK1 or vector control and treated with AZD6244 for 24h. Cells were collected for apoptosis assay. **(B)** Quantification of **(A)**. Data represented as mean ± SD (n = 3). **(C)** Apoptosis assay of HCT116 and SW480 infected with shRNA targeted with PLK1 or vector control treated with GSK for 24h. **(D)** A549 and CFPAC-1 were infected with shRNAs targeted with PLK1 or vector control, then treated with AZD6244 for 24h. Cells were collected for apoptosis assay. **(E and F)** SW480 were treated with DMSO, AZD6244, BI-2536, or their combination for 48h. Cells were collected for apoptosis assay. **(F)** Quantification of **(E)**. Data represented as mean ± SD (n = 3). **(G)** Effects of BI-2536 on cell apoptosis in SW480 cells expressing vector or shRNA targeting GRB7. **(H-J)** Effects of PLK1 knockdown on cell proliferation in vector or GRB7 expressing-HCT116 and SW480 cells. **(I)** Quantification of HCT116, **(J)** Quantification of SW480. ∗*p* < 0.05, ∗∗*p* < 0.01, ∗∗∗*p* < 0.001, or ∗∗∗∗*p* < 0.0001.

**Figure S8**


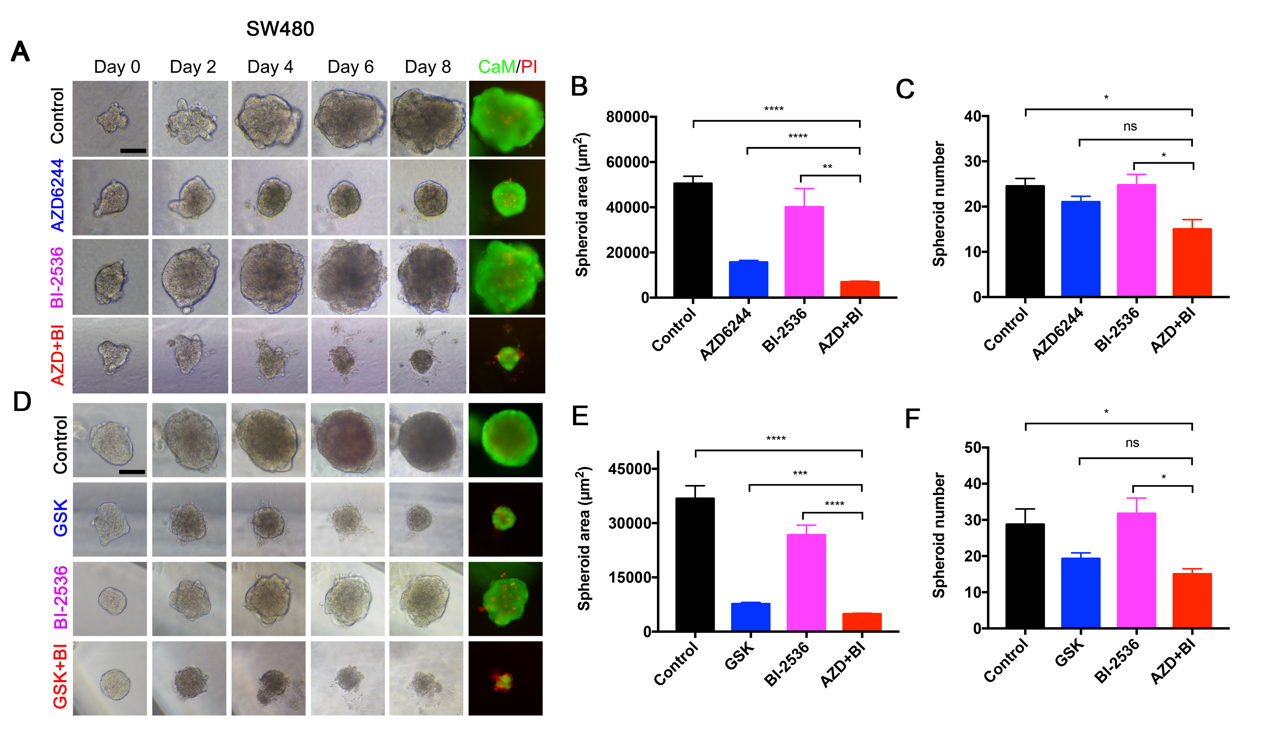


**Figure S8 Combination inhibition of PLK1 and MEK suppressed cell growth in 3D colorectal cancer cell culture system**

**(A-C)** Representative phase-contrast and immunofluorescence staining images of SW480 treated with DMSO, AZD6244, BI-2536, or their combination for 8 days in 3D culture system are shown (CaM: green; PI: red). Scale bar, 100 μM. **(B)** Quantification of spheroid area. **(C)** Quantification of spheroid number. **(D-F)** Representative phase-contrast and immunofluorescence staining images of SW480 treated with DMSO, GSK, BI-2536, or their combination for 8 days in 3D culture system are shown (CaM: green; PI: red). Scale bar, 100 μM. **(E)** Quantification of spheroid area. **(F)** Quantification of spheroid number. ∗*p* < 0.05, ∗∗*p* < 0.01, ∗∗∗*p* < 0.001, or ∗∗∗∗*p* < 0.0001.

**Figure S9**

**
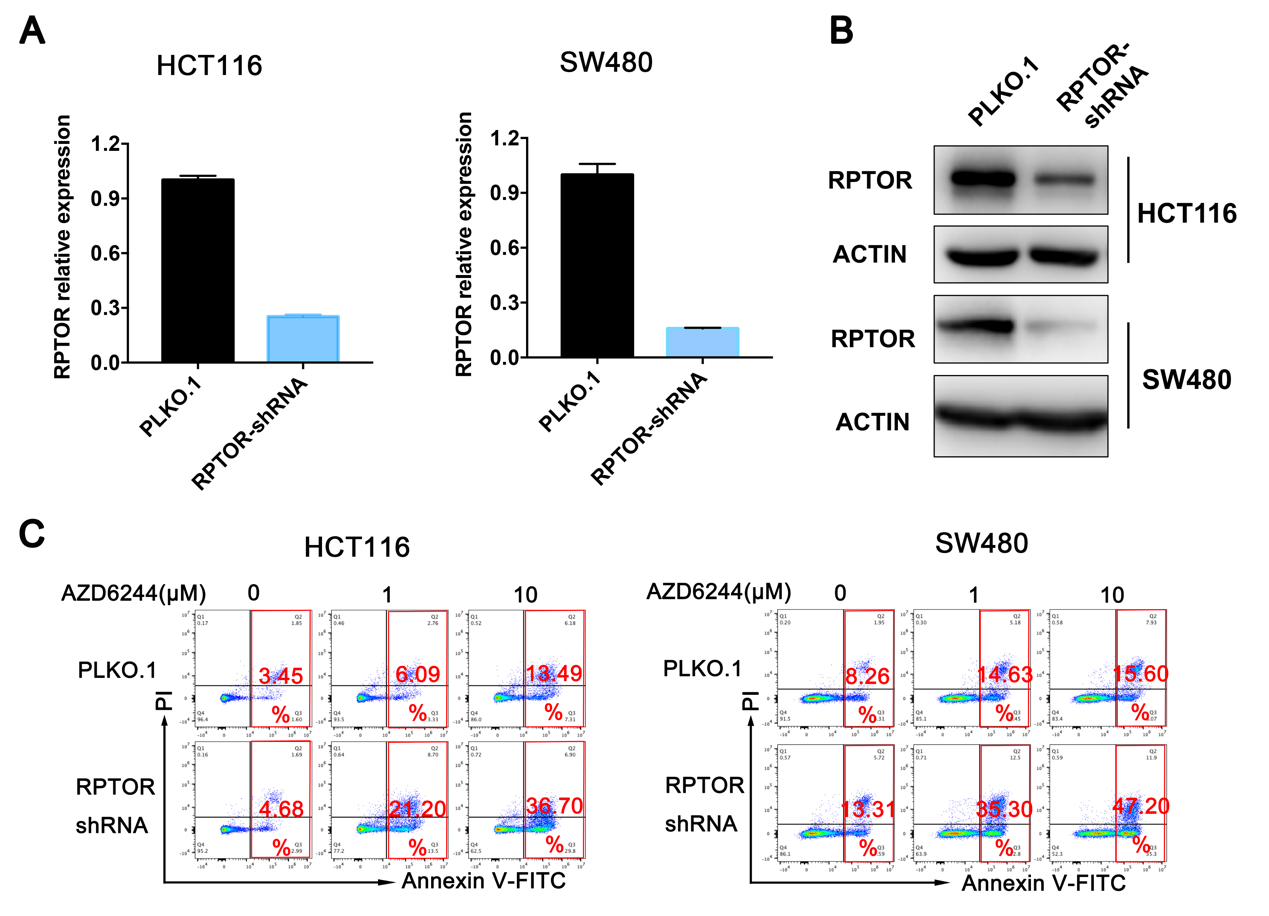
**

**Figure S9 Combination inhibition of RPTOR and MEK promoted cell apoptosis**

**(A)** qPCR analysis of RPTOR expression in HCT116 and SW480 infected with shRNAs targeting RPTOR or vector control. Data represent mean ± SD (n = 3). **(B)** HCT116 and SW480 were infected with shRNAs targeting RPTOR or vector control. Cell lysates were made for immunoblot analysis. **(C)** HCT116 and SW480 were infected with shRNAs targeted with RPTOR or vector control and treated with AZD6244 for 24h. Cells were collected for apoptosis assay.

**Reference**

1. Szlachta K, Kuscu C, Tufan T, Adair SJ, Shang S, Michaels AD, et al. CRISPR knockout screening identifies combinatorial drug targets in pancreatic cancer and models cellular drug response. Nature communications. 2018;9(1):4275.

2. Varshney GK, Carrington B, Pei W, Bishop K, Chen Z, Fan C, et al. A high-throughput functional genomics workflow based on CRISPR/Cas9-mediated targeted mutagenesis in zebrafish. Nature protocols. 2016;11(12):2357-75.

3. Liu Y, Liu K, Yin L, Yu Y, Qi J, Shen W-H, et al. H3K4me2 functions as a repressive epigenetic mark in plants. Epigenetics & Chromatin. 2019;12(1):40.

4. Yu C, Zhang M, Song J, Zheng X, Xu G, Bao Y, et al. Integrin-Src-YAP1 signaling mediates the melanoma acquired resistance to MAPK and PI3K/mTOR dual targeted therapy. Molecular Biomedicine. 2020;1(1):12.

5. Fedele C, Ran H, Diskin B, Wei W, Jen J, Geer MJ, et al. SHP2 Inhibition Prevents Adaptive Resistance to MEK Inhibitors in Multiple Cancer Models. Cancer discovery. 2018;8(10):1237-49.

6. Liu X, Feng Y, Xu G, Chen Y, Luo Y, Song J, et al. MAPK-Targeted Drug Delivered by a pH-Sensitive MSNP Nanocarrier Synergizes with PD-1 Blockade in Melanoma without T-Cell Suppression. 2019;29(12):1806916.
